# Single-cell transcriptomics reveals skewed cellular communication and phenotypic shift in pulmonary artery remodeling

**DOI:** 10.1172/jci.insight.153471

**Published:** 2022-10-24

**Authors:** Slaven Crnkovic, Francesco Valzano, Elisabeth Fließer, Jürgen Gindlhuber, Helene Thekkekara Puthenparampil, Maria Basil, Mike P. Morley, Jeremy Katzen, Elisabeth Gschwandtner, Walter Klepetko, Edward Cantu, Heimo Wolinski, Horst Olschewski, Jörg Lindenmann, You-Yang Zhao, Edward E. Morrisey, Leigh M. Marsh, Grazyna Kwapiszewska

**Affiliations:** 1 Ludwig Boltzmann Institute for Lung Vascular Research, Graz, Austria.; 2 Division of Physiology & Pathophysiology, Otto Loewi Research Center and; 3 Diagnostic and Research Institute of Pathology, Diagnostic and Research Center of Molecular BioMedicine, Medical University of Graz, Graz, Austria.; 4 Penn Center for Pulmonary Biology, Department of Medicine, University of Pennsylvania, Philadelphia, Pennsylvania, USA.; 5 Department of Thoracic Surgery, Medical University of Vienna, Vienna, Austria.; 6 Department of Surgery, University of Pennsylvania, Philadelphia, Pennsylvania, USA.; 7 Institute of Molecular Biosciences and; 8 Field of Excellence BioHealth, University of Graz, Graz, Austria.; 9 Division of Pulmonology, Department of Internal Medicine; and; 10 Division of Thoracic and Hyperbaric Surgery, Department of Surgery, Medical University of Graz, Graz, Austria.; 11 Program for Lung and Vascular Biology, Section of Injury Repair and Regeneration, Stanley Manne Children’s Research Institute, Ann & Robert H. Lurie Children’s Hospital of Chicago, Chicago, Illinois, USA.; 12 Departments of Pediatrics, Pharmacology, and Medicine, Feinberg School of Medicine, Northwestern University, Chicago, USA.; 13 Institute of Lung Health, German Center for Lung Research (DZL), Giessen, Germany.

**Keywords:** Pulmonology, Vascular Biology, Hypertension

## Abstract

A central feature of progressive vascular remodeling is altered smooth muscle cell (SMC) homeostasis; however, the understanding of how different cell populations contribute to this process is limited. Here, we utilized single-cell RNA sequencing to provide insight into cellular composition changes within isolated pulmonary arteries (PAs) from pulmonary arterial hypertension and donor lungs. Our results revealed that remodeling skewed the balanced communication network between immune and structural cells, in particular SMCs. Comparative analysis with murine PAs showed that human PAs harbored heterogeneous SMC populations with an abundant intermediary cluster displaying a gradient transition between SMCs and adventitial fibroblasts. Transcriptionally distinct SMC populations were enriched in specific biological processes and could be differentiated into 4 major clusters: oxygen sensing (enriched in pericytes), contractile, synthetic, and fibroblast-like. End-stage remodeling was associated with phenotypic shift of preexisting SMC populations and accumulation of synthetic SMCs in neointima. Distinctly regulated genes in clusters built nonredundant regulatory hubs encompassing stress response and differentiation regulators. The current study provides a blueprint of cellular and molecular changes on a single-cell level that are defining the pathological vascular remodeling process.

## Graphical abstract

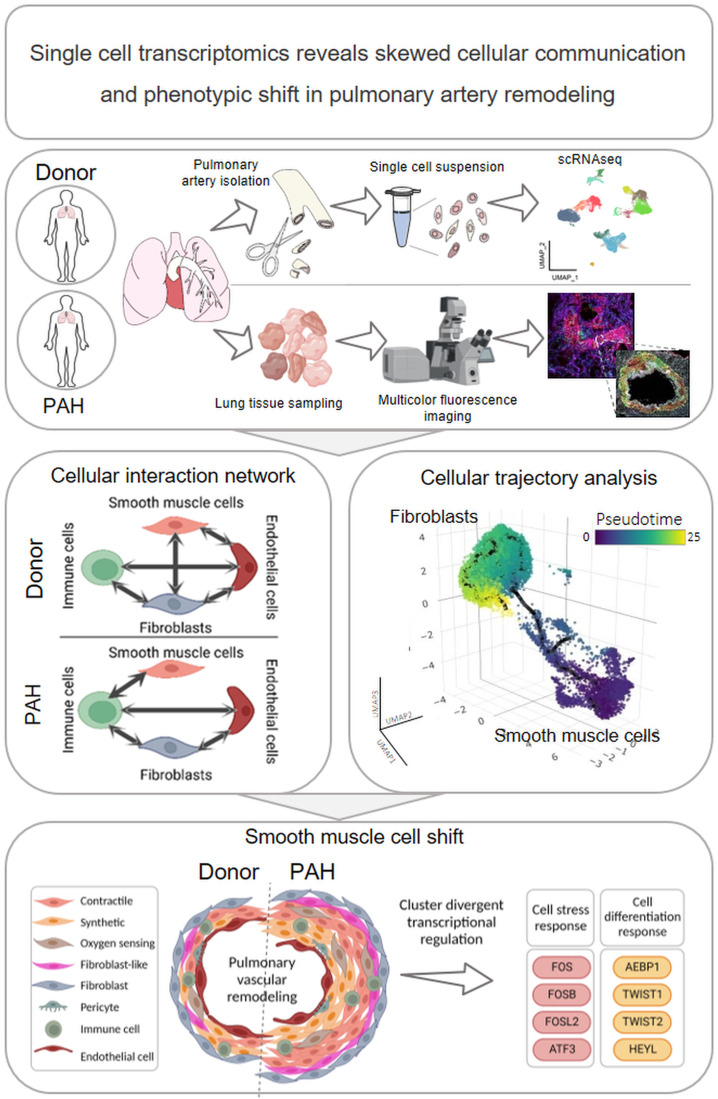

## Introduction

Pulmonary vascular remodeling represents a major therapeutic challenge in the care of patients with pulmonary hypertension (PH) (1). PH can manifest as a rare isolated disease of pulmonary vessels (such as idiopathic pulmonary arterial hypertension, IPAH) or more commonly accompanying chronic heart or chronic lung diseases (2, 3). Morphological hallmarks of this disease include thickening of all 3 vessel layers (intima, media, and adventitia) with concomitant partial or complete lumen obstruction (4–6). Contributors to vascular remodeling include increased cellularity, deposition of extracellular matrix (ECM), and an altered immune cell profile (5, 7). In particular, advanced remodeling is characterized by the appearance of a neointima containing cells expressing α–smooth muscle actin 2 (*ACTA2*) (8, 9). However, neointimal cells show weak or even no expression of other classical smooth muscle cell (SMC) markers (10), which raises questions into their origin and function. Furthermore, *ACTA2* expression is not exclusive for SMCs, as some fibroblasts express diverse *ACTA2* levels (11, 12), while other cell types, such as endothelial cells, can express *ACTA2* following in vitro stimulation (13). To date, it is still unclear whether cells within the neointima represent a unique variation of vascular SMCs or a distinct pathogenic lineage arising from a transdifferentiation event (14, 15).

Under normal conditions, *ACTA2*-expressing cells within the medial layer represent classical pulmonary artery smooth muscle cells (PASMCs) and fulfill several effective roles, such as providing structural support or vasoactive responses (16). Landmark studies investigating the development of the pulmonary arteries (PAs) have shown that PASMC maturation is accompanied by increasing expression of contractile proteins, such as calponin (*CNN*), smoothelin (*SMTN*), and smooth muscle myosin heavy chain (*MYH*), and is termed the contractile phenotype (17, 18). In contrast, the synthetic phenotype possesses higher expression of ECM proteins and ECM-processing enzymes (19, 20). Based on murine fate mapping approaches, it has been postulated that PAs harbor a distinctive pathogenic PASMC population that is proliferation privileged and undergoes clonal expansion upon external cues (8, 14). However, it is unknown whether a similar pathogenic PASMC population exists in human PAs.

To date, uncovering the heterogeneity of PASMCs, in health and disease, has been hindered by the limited number of cell population markers or the need for in vitro subculturing on plastic (21). Therefore, the identification of physiologically and pathologically relevant SMC populations has been potentially overlooked. To overcome these barriers, we adopted an unbiased single-cell transcriptomics approach to investigate the cellular composition and heterogeneity in both healthy and remodeled PAs. A high-resolution map of human PA cellularity revealed extensive presence of diverse immune cells, homogenous fibroblasts, and intrinsic heterogeneity in SMC clusters. We observed 4 major PASMC clusters, which were conserved in human coronary arteries but less so in murine PAs. Remodeled PAs displayed a striking shift from normally balanced intercellular communication pattern toward a PASMC-fibroblast–centered signaling hub. Cumulatively, our results indicate that vascular remodeling associated with adult human pulmonary vascular disease is centered on PASMC phenotypic shift and skewed intercellular signaling pattern.

## Results

### Complex cellular composition of the PA compartment.

**Figure 1:**
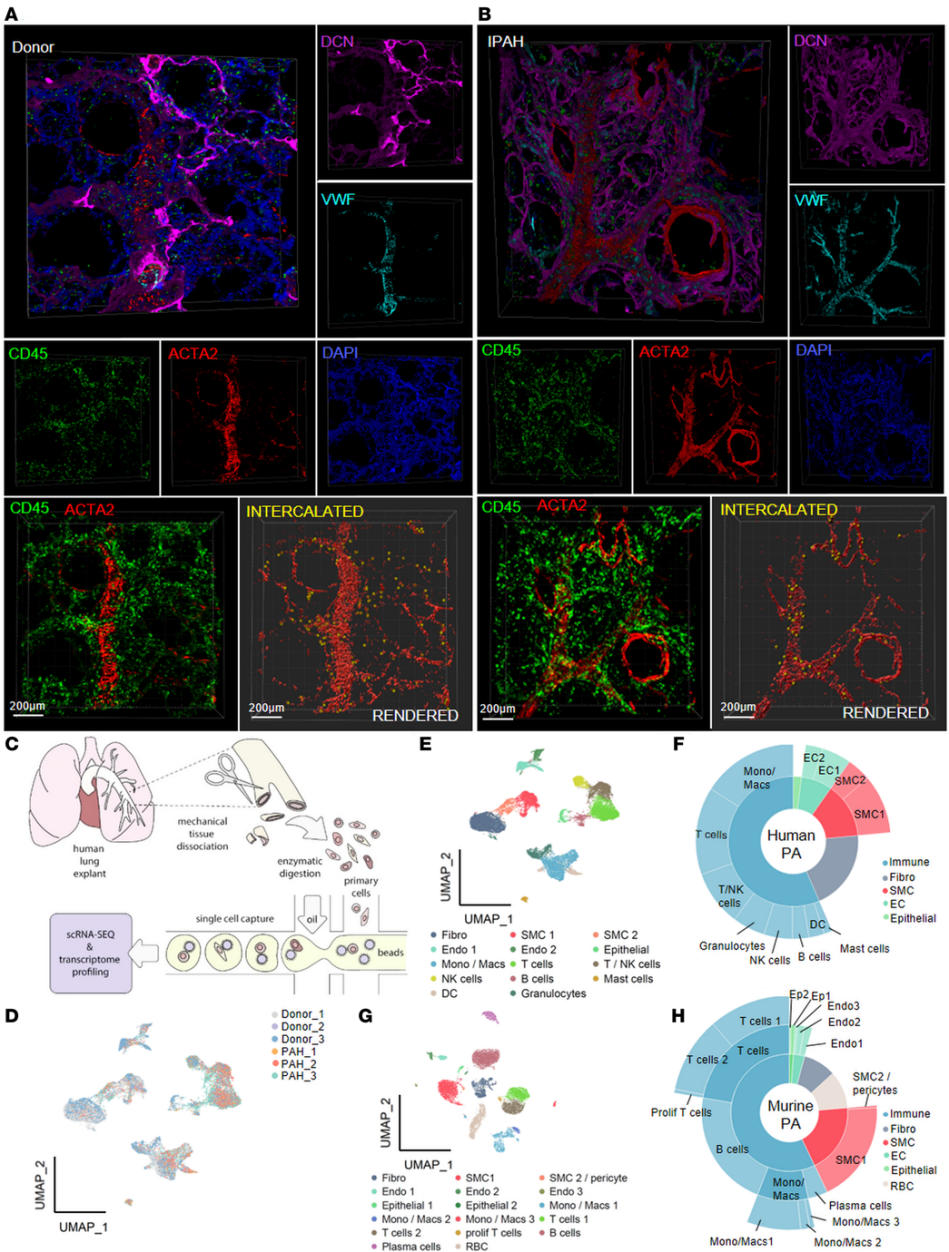
Pulmonary artery niche is composed of diverse structural and immune cell populations. (**A** and **B**) Representative 3-dimensional (3D) images of precision-cut human lung slices (200 μm thickness, *n* = 5) from donor (**A**) and idiopathic pulmonary arterial hypertension (IPAH) patient (**B**) stained against endothelial cells (V*WF*, cyan), smooth muscle cells (*ACTA2*, red), fibroblasts (*DCN*, magenta), and immune cells (*CD45*, green). DAPI, nuclei (blue, top panel). 3D rendering of the smooth muscle cells (red) and immune cells embedded in the arterial wall (yellow). Scale bar = 200 μm. (**C**) Scheme of human pulmonary artery (PA) processing for single-cell RNA sequencing (scRNA-Seq). (**D**) Uniform manifold approximation and projection (UMAP) of the PA scRNA-Seq data of donor (*n* = 3) and pulmonary arterial hypertension (PAH) samples (*n* = 3). (**E**) Annotated UMAP of human PA scRNA-Seq. (**F**) Sunburst plot representing human PA cell populations’ proportions. (**G**) Annotated UMAP of scRNA-Seq captured from normoxia (*n* = 3) and 3 weeks’ hypoxia (*n* = 3) murine PAs. (**H**) Sunburst plot representing murine PA cell populations’ proportions. Fibro, fibroblasts; SMC1, smooth muscle cells 1; SMC 2/pericyte, smooth muscle cells 2 and pericytes; Endo 1,2,3/EC1,2, endothelial cells 1,2,3; Epithelial 1,2/Epit1,2, epithelial cells 1,2; Mono/Macs 1,2,3, monocytes and macrophages 1,2,3; prolif T cells, proliferating T cells; RBC, red blood cells; DC, dendritic cells.

To gain detailed insight into the 3-dimensional (3D) organization of human PAs, we applied multicolor immunofluorescence staining with confocal imaging of precision-cut human lung tissue sections. Staining against endothelial cell (EC), SMC, fibroblast, and immune cell markers (von Willebrand factor/*VWF*, *ACTA2*, decorin/*DCN*, and *CD45*, respectively), revealed the expansion of SMC coverage toward the distal PA vascular tree in IPAH lungs (Figure 1, A and B). In both normal and remodeled vessels, there was a prominent abundance of immune cells not only in the perivascular/adventitial region but also within the vascular wall (Figure 1, A and B).

We next performed an unbiased assessment of cellular heterogeneity by applying single-cell RNA (scRNA) sequencing (scRNA-Seq) (Figure 1C). A total of 29,069 cells were sequenced from 3 donor and 3 pulmonary arterial hypertension (PAH) PAs, with a median of 1,015 genes and 2,759 unique molecular identifiers (UMIs) per cell. The scRNA transcriptome from all samples was concatenated and analyzed as a single data set. After quality control 22,704 cells (11,759 donor, 10,945 PAH) were retained. Dimension reduction, clustering, and visualization via uniform manifold approximation and projection (UMAP) of the scRNA expression data set yielded good overlap of all samples (Figure 1D and Supplemental Figure 1, A and B; supplemental material available online with this article; https://doi.org/10.1172/jci.insight.153471DS1). Gene enrichment analysis identified 14 distinct cell clusters. The top 10 enriched genes for each cluster and the corresponding expression profiles are depicted in Supplemental Figure 1, D and E. Manual cluster annotation based on comparison of cluster-identifying genes and classical cell type markers resulted in 2 SMC, 2 endothelial, 1 fibroblast, and 8 immune cell clusters (Figure 1, E and F; Supplemental Figure 1C; and Supplemental Table 1).

In a parallel approach we performed a single-cell capture and RNA-sequencing experiment from 3 murine PAs from normoxic condition and 3 PAs dissected from mice exposed to 3 weeks of normobaric hypoxia (Supplemental Figure 2, A and B). Manual annotation of 17 resulting clusters identified 2 SMC, 3 endothelial, 1 fibroblast, and 8 immune cell clusters (Figure 1, G and H, and Supplemental Figure 2, A–E).

**Figure 2:**
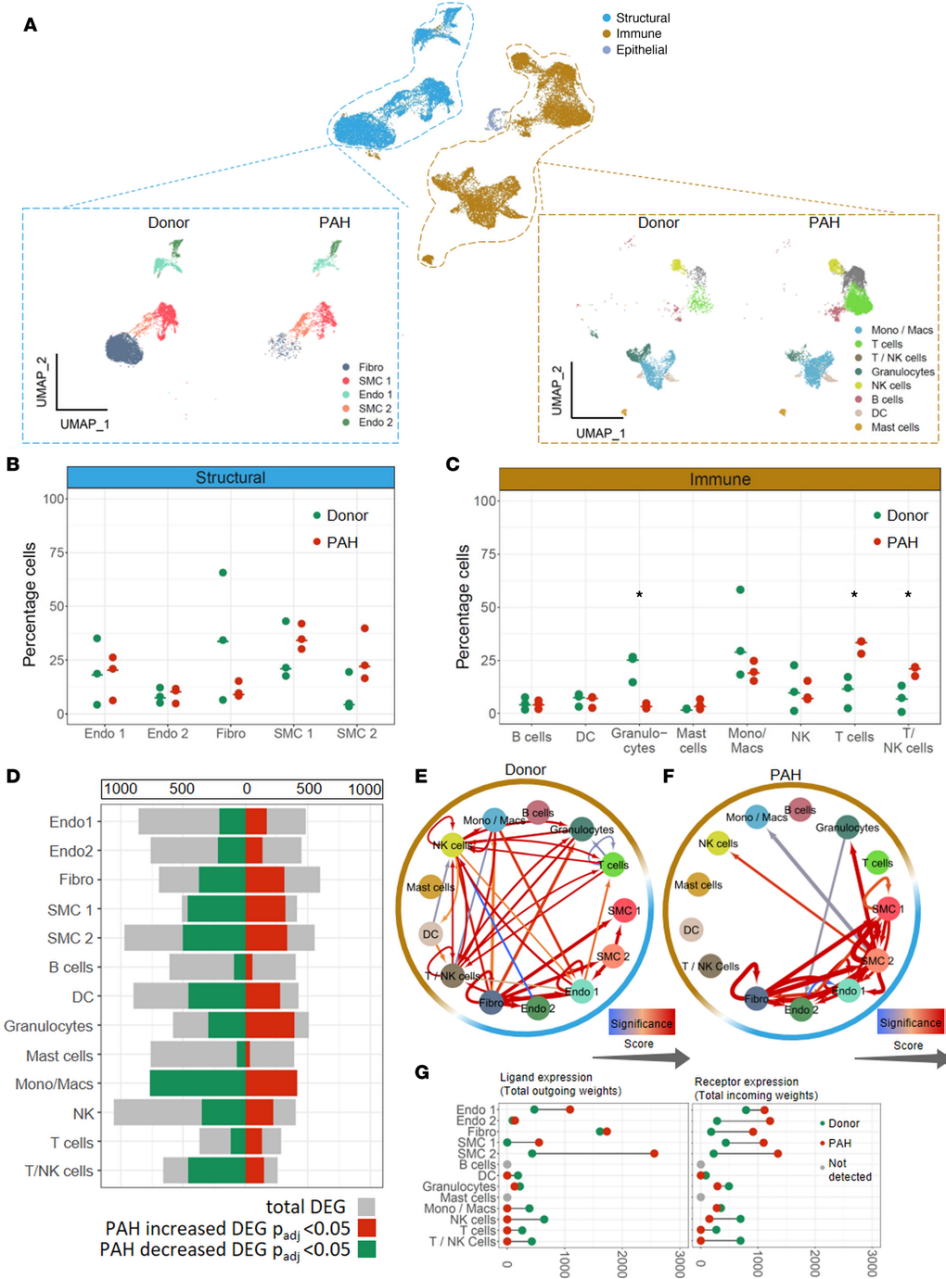
Vascular remodeling alters intercellular signaling in human pulmonary artery. (**A**) Uniform manifold approximation and projection (UMAP) of pulmonary artery (PA) single-cell RNA sequencing (scRNA-Seq) from donors (*n* = 3) and pulmonary arterial hypertension (PAH) patients (*n* = 3) annotated and subdivided into structural, immune, and epithelial cell types. Endo 1,2, endothelial cells 1,2; Fibro, fibroblasts; SMC 1,2, smooth muscle cells 1,2; DC, dendritic cells; Mono/Macs, monocytes and macrophages. (**B** and **C**) Cell population percentage of (**B**) structural and (**C**) immune cell types in donor (green) and PAH (red) samples. *T* test, *P < 0.05. (**D**) Bar plot representing differentially expressed gene (DEG) analysis performed on PA cell populations. Wilcoxon rank sum test with Bonferroni adjustment, *P* < 0.05. Gray bars indicate the total number of DEG terms highlighted from the analysis while colored part indicates the proportion of DEG terms significantly enriched in PAH (red) and donor (green). Ligand-receptor interactions performed on (**E**) donor and (**F**) PAH PA structural and immune cell types using FANTOM5 project with added weights according to STRING database. Thickness of arrows is relative to total number of found interaction pairs; color-coding depicts gradient of significant interactions. Permutation test (number of permutations = 100,000), *P* < 0.05. (**G**) Scheme representing differential expression of ligand-receptor pairs in donor (green) and PAH (red) PA. Gray dots represent ligands or receptors not detected in the analysis. Score assessed as the sum of the total ligand or receptor weights associated with every cell population.

To highlight the changes in relative cell number abundance, we separated our human scRNA data set into structural and immune cell subsets and subsequently performed pairwise comparison of each cell type in healthy and diseased status (Figure 2, A–C). This comparison supported previously reported shifts in relative abundance of major cell populations, such as increased trend of SMC proportions (Figure 2B), T cells, and T/NK cells (Figure 2C), as well as decrease of granulocytes (Figure 2C) in remodeled vessels (7, 9). Beyond perturbations in cellular abundance, vascular remodeling is associated with extensive transcriptomic changes (22). However, until the advent of scRNA analysis, it was impossible to differentiate between transcriptomic changes arising from altered cellular composition and those due to cell type–specific differential gene expression. We therefore performed a cluster-based analysis of differentially expressed genes (DEGs) between donor and PAH samples for each of the 14 clusters (Figure 2D). Noticeably, the majority of the transcriptome changes were detected within SMC1, SMC2, and certain immune cell clusters. In a further analysis focusing on cellular interactions, we built a cell interactome depicting cell-to-cell communications based on weighted expression of ligand-receptor pairs between different cluster pairs (Supplemental Table 2). In healthy PAs, interactions were spread evenly among different clusters and showed balanced interconnections between the structural and immune cell populations (Figure 2E). However, PAH samples showed a severe distortion of the normal signaling landscape, with the majority of cellular interactions shifted toward structural cells, in particular SMC and fibroblast clusters (Figure 2F), as displayed by the increase of structural ligand-receptor interaction weights and the decrease of immune ligand-receptor interaction weights (Figure 2G and Supplemental Tables 2 and 3).

### Mural cell composition in human PAs.

**Figure 3:**
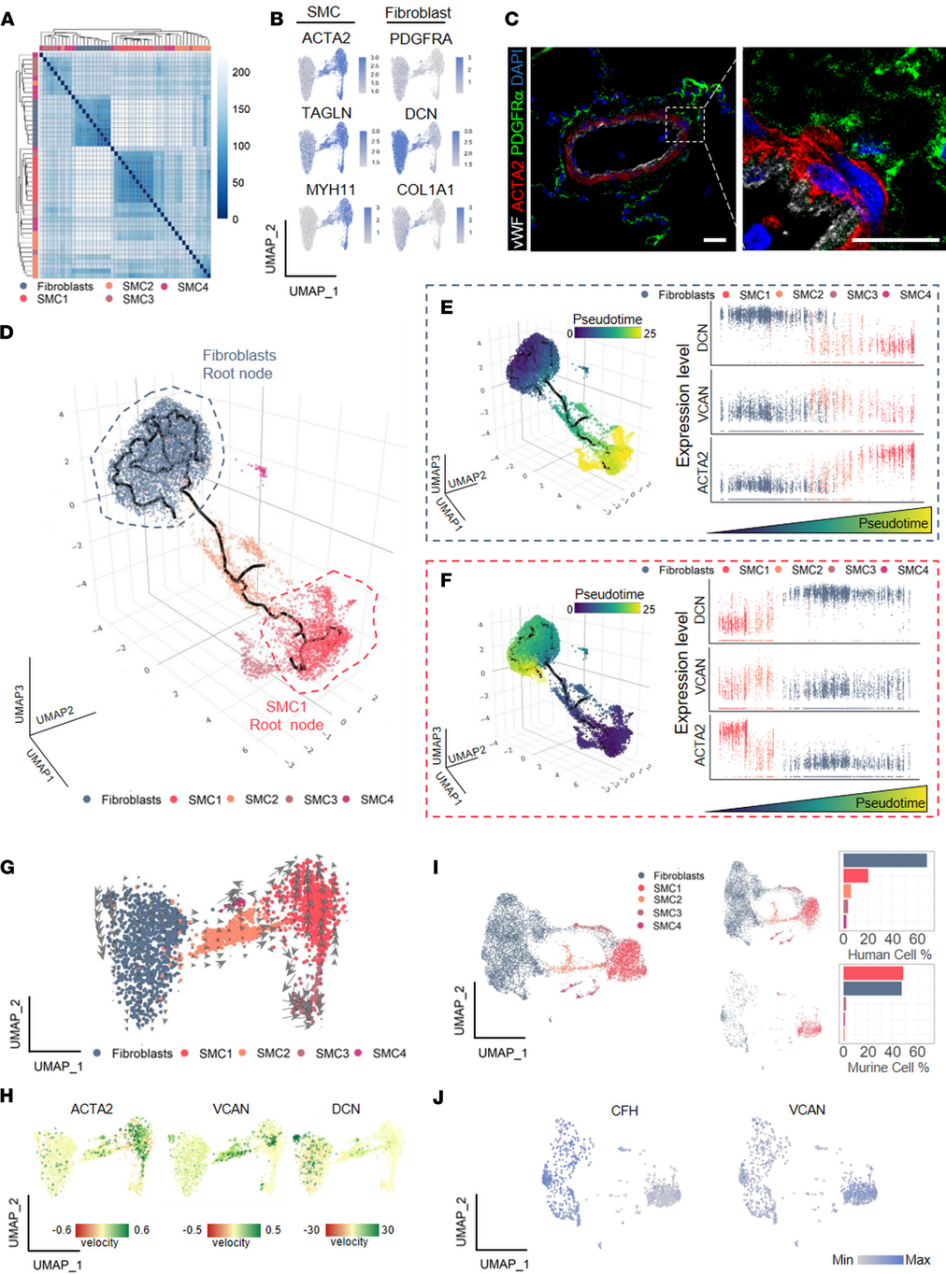
Human pulmonary artery possesses a specific smooth muscle cell–fibroblast intermediary cluster. (**A**) Hierarchical clustering heatmap of top 10 genes enriched in smooth muscle cell (SMC) and fibroblast clusters. Wilcoxon rank sum test with Bonferroni adjustment, *P* < 0.05 and |log_2_(fold change)| > 0.25. (**B**) Uniform manifold approximation and projection (UMAP) expression plots of *ACTA2*, *TAGLN*, *MYH11* and *PDGFRA*, and *DCN* and *COL1A1*. The color gradient represents the average expression across the fibroblasts and SMC clusters. (**C**) Representative immunofluorescence staining of *ACTA2* (red, medial layer), *PDGFRA* (green, adventitial layer), *VWF* (gray, intima layer), and DAPI (blue, nuclei) in human formalin-fixed, paraffin-embedded (FFPE) lung tissue. Scale bar = 20 μm (*n* = 5 vessels). (**D**) Trajectory inference overlaid on 3-dimensional (3D) UMAP of the extracted SMC and fibroblast clusters. (**E** and **F**) Color-coded pseudotime calculation overlaid on 3D UMAP of the extracted SMC and fibroblast subset using fibroblasts (**E**) and SMC1 (**F**) as root nodes (left panel). Scatterplots illustrating the different expression of canonical markers for fibroblasts (*DCN*), SMC2 (*VCAN*), and SMC (*ACTA2*) going along with the increase of pseudotime along the trajectory from fibroblast to SMC1 (**E**, right panel) or from SMC1 to fibroblast (**F**, right panel). (**G**) RNA velocity overlaid on UMAP of the extracted SMC and fibroblast subset. RNA velocity analysis identified 317 velocity genes across the data set. (**H**) RNA velocity of canonical markers for SMC (*ACTA2*), SMC2 (*VCAN*), and fibroblasts (*DCN*). (**I**) Integration of human and murine pulmonary artery single-cell RNA-Seq data set, showing the extracted fibroblast-SMC subset (left) with associated bar plot depicting cell composition (Fibroblasts, SMC1,2,3,4, right) in the 2 data sets. (**J**) UMAP expression plots of *CFH* and *VCAN* in integrated human and mouse fibroblast-SMC data set. The color gradient represents the average expression across the extracted fibroblast and SMC subset.

Due to the central role of SMC-fibroblast clusters in the intercellular signaling network of remodeled vessels, we focused our analysis on these cellular components. All cells within the fibroblast and SMC clusters were extracted from the PA data set and subsequently reclustered. This gave 5 clusters with distinct expression profiles (Figure 3A). Classical genes for SMCs (*ACTA2*, *TAGLN*, *MYH11*) and fibroblasts (*PDGFRA*, *DCN*, *COL1A1*) were used to perform cluster annotation (Figure 3B), which resulted in the following classification: 1) fibroblasts, 2) SMC (SMC1 and SMC3), and 3) an intermediary group of cells having a shared expression profile between SMCs and fibroblasts (SMC2 and SMC4) (Supplemental Table 4). Representative staining of classical fibroblast (*PDGFRA*) and SMC (*ACTA2*) lineage markers (9, 11) verified expected localization in adventitial fibroblasts (in the outer layer of PAs, distal of external elastic lamina) and SMCs (medial localization), respectively, but also highlighted low occurrence of double-positive cells (Figure 3C). To identify markers that could better discriminate between adventitial fibroblasts and SMCs, we performed differential gene expression analysis between the fibroblast cluster and all SMC clusters. We identified the top 18 genes enriched in the fibroblast cluster and verified the fibroblast-enriched expression of these markers on the entire PA data set (Supplemental Figure 3, A–D). We observed that *SERPINF1*, *SLPI*, *C3*, and *MGST1* were good discriminatory markers between fibroblasts and SMCs, while *C3* and *CTSK* might have good discriminatory potential toward fibroblasts in the global context of PA cells (Supplemental Figure 3, A–D).

The gradient expression of both SMC and fibroblast markers in 2 intermediary clusters (SMC2, SMC4) raised the question of the origin of these cells. Trajectory inference and pseudotime calculation were used as unbiased analysis to gauge whether these intermediary clusters are more similar, and thus derived, from fibroblasts or SMCs. By positioning transcriptionally similar cells close together and mapping them along a putative differentiation trajectory, all clusters were arranged on a fibroblast/myocyte axis with classical fibroblasts on the one end and classical SMCs (SMC1) on the other (Figure 3D). Next, distances between cells were compared by calculating pseudotime score for each cell, using either fibroblasts (Figure 3E and Supplemental Figure 3, E and F) or SMC1 (Figure 3F and Supplemental Figure 3, E and F) as the putative origin. In both cases, SMC2 were closely aligned with classical SMC (SMC1) and SMC4 with fibroblasts (Figure 3, E and F). As a quality control, we mapped the expression profile of 3 markers enriched within fibroblast, classical SMC, and intermediary clusters (*DCN*, *ACTA2*, *VCAN*, respectively), as a function of pseudotime (Figure 3, E and F). Expression of these markers was in line with trajectory inference and pseudotime scoring, with *DCN* and *ACTA2* enriched in fibroblasts and SMC1, respectively (Figure 3, E and F). Intermediary cluster SMC2 displayed less expression of *ACTA2*, enrichment in *VCAN*, and low *DCN* expression (Figure 3, E and F). In contrast, SMC4 was characterized by high *DCN* expression (Figure 3, E and F). To conclusively determine directionality and assign cluster origins, we performed transcriptional kinetics analysis for each single cell using RNA velocity (Figure 3G). The results show 2 distinct routes originating from SMC1 cluster and directed toward SMC2 and SMC3, suggesting that these 2 clusters are derived from SMC1. SMC4, however, displayed bidirectional kinetics and positive scores for both *ACTA2* and *DCN* velocities (Figure 3, G and H). This suggests that SMC4 represent a mixed population derived from fibroblasts acquiring an SMC phenotype and from SMCs acquiring a fibroblast phenotype.

As most insight into fibroblast-SMC distinction and SMC populations was based on previous lineage tracing approaches in murine models (8, 9, 11), we questioned whether a similar heterogeneity in the PA medial layer can be observed in murine PAs. We combined human and mouse scRNA-Seq data, extracted SMC and fibroblast clusters, and reanalyzed the extracted cells (Figure 3I and Supplemental Figure 3, G and H). In the murine data set, most cells belonged to either the SMC1 or fibroblast cluster, followed by a small percentage of SMC3 cluster–like cells (Figure 3I). Cells related to human intermediary clusters, SMC2 and SMC4, represented only rare events in murine PAs (Figure 3I). Interestingly, the 2 top marker genes for human SMC2 cluster, *CFH* and *VCAN*, were contained in the murine data set within either the fibroblast or classical SMC1 cluster, respectively (Figure 3J).

**Figure 4:**
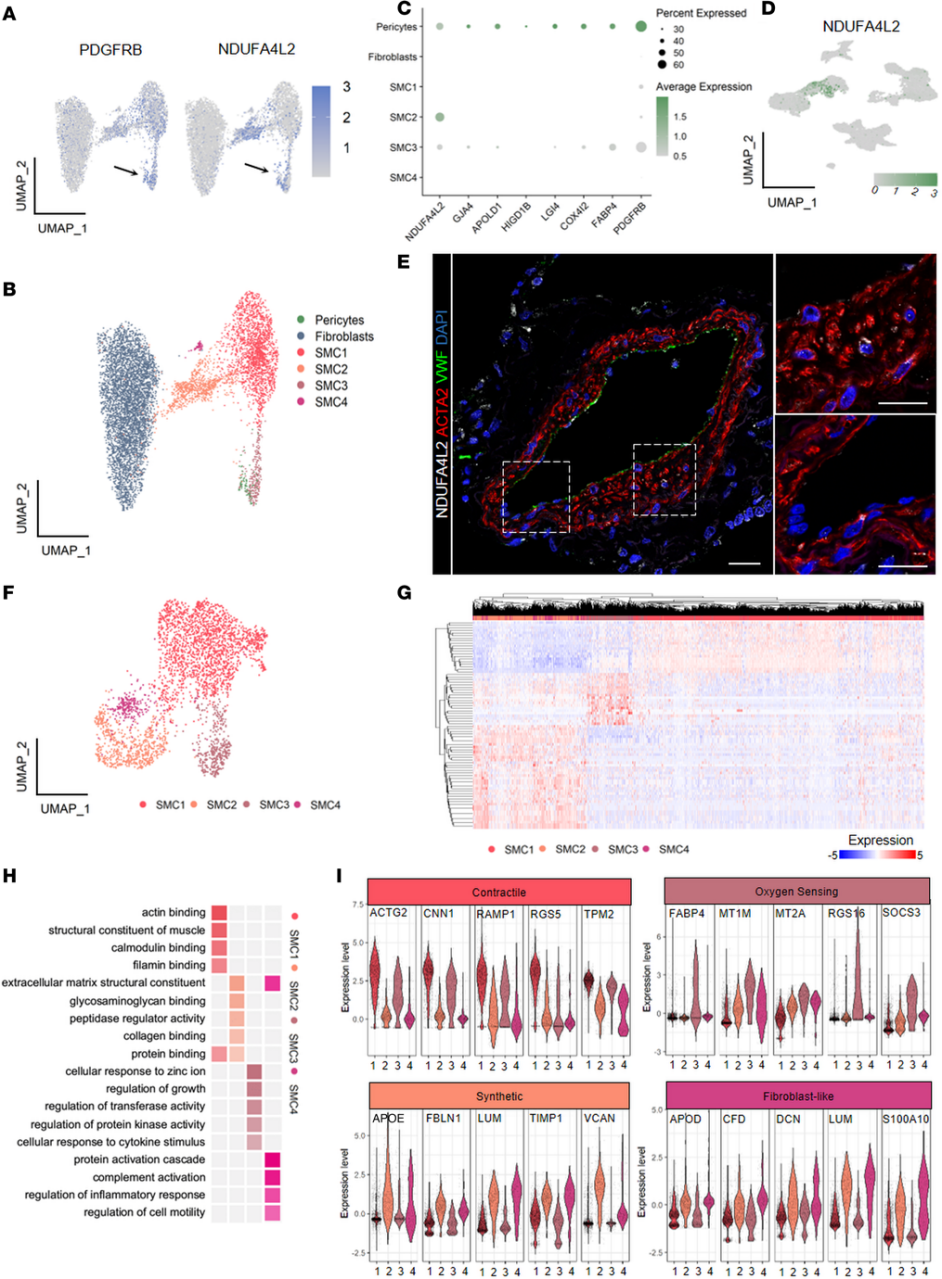
Pulmonary artery pericytes represent a minor group of *ACTA2*-positive cells. (**A**) Uniform manifold approximation and projection (UMAP) expression plots of *PDGFRB* and *NDUFA4L2*. Color gradient represents the average expression across the fibroblasts and smooth muscle cell (SMC) clusters. (**B**) UMAP of the extracted fibroblast, SMC, and pericyte subsets. (**C**) Dot plot of the top 8 genes from the pericyte cluster (*NDUFA4L2*, *GJA4*, *APOLD1*, *HIGD1B*, *LGI4*, *COX4I2*, *FABP4*, and *PDGFRB*). Dot size represents percentage of cells expressing the gene; color gradient represents the average expression across the data set. (**D**) UMAP gene expression plot of *NDUFA4L2*. The color gradient represents the average expression across the entire pulmonary artery (PA) data set. (**E**) Representative immunofluorescence staining of the expression of *NDUFA4L2*-positive cells (in gray) embedded in different locations of the PA medial layer in human formalin-fixed, paraffin-embedded (FFPE) lung tissue (*ACTA2*, red; *VWF*, green; and DAPI, blue). Scale bar = 20 μm (*n* = 4). (**F**) UMAP of the extracted SMC subset resulting in 4 subclusters (SMC1,2,3,4, smooth muscle cell 1,2,3,4). (**G**) Hierarchical clustering heatmap of the top 50 marker genes across the SMC subset. Wilcoxon rank sum test with Bonferroni adjustment, *P* < 0.05. (**H**) Gene Ontology (GO) analysis performed on cluster-enriched genes. Fisher’s exact test with Benjamini-Hochberg adjustment, *P* < 0.05. (**I**) GO-based new nomenclature of the 4 SMC clusters (contractile, oxygen sensing, synthetic, fibroblast-like) with associated violin plot of 5 most enriched genes per cluster (*ACTG2*, *CNN1*, *RAMP1*, *RGS5*, *TPM2* for contractile; *APOE*, *FBLN1*, *LUM*, *TIMP1*, *VCAN* for synthetic; *FABP4*, *MT1M*, *MT2A*, *RGS16*, *SOCS3* for oxygen sensing; *APOD*, *CFD*, *DCN*, *LUM*, *S100A10* for fibroblast-like).

Surprisingly, the analysis of our data set did not highlight the presence of a distinct cluster with a characteristic fingerprint indicative of pericytes in human PAs. This could be explained by rare pericyte abundance or indicate high transcriptional similarity to SMCs. Plotting global expression of classical pericyte markers *PDGFRB*, *CSPG4*, and *RGS5* revealed their dispersed expression across different clusters of PA-resident populations (Supplemental Figure 4A). Analyzing additional markers retrieved from PanglaoDB (23) further supported the distribution of pericyte markers throughout the fibroblast and SMC clusters (Supplemental Figure 4B). However, closer examination of *PDGFRB* and *NDUFA4L2*, as well as other pericyte-specific markers such as *CSPG4* and *RGS5* (Supplemental Figure 4C) in the fibroblast and SMC subsets, showed enrichment within 1 *ACTA2*^hi^ cluster (SMC3; Figure 4A). Manual subsetting of this potential subcluster (Figure 4B) and consequent DEG analysis revealed it may indeed represent pericytes. The top 8 DEGs within this putative pericyte cluster confirmed enriched expression of potentially novel pericyte markers, alongside classical markers such as *PDGFRB* (Figure 4C). The expression profile of *NDUFA4L2* appeared rather restricted and specific to pericyte populations (Figure 4D). Staining for *NDUFA4L2* confirmed on one hand subendothelial localization and coexpression of this protein with *ACTA2*, in line with pericyte definition, but also displayed an additional labeling of medial *ACTA2* cells (Figure 4E). Together, these results point to a very high transcriptional similarity of pericytes and classical SMCs, with localization within the PA vessel wall still being the most conclusive pericyte characteristic. Furthermore, the close localization of pericytes and other SMCs to the EC layer can erroneously assign SMC characteristics to ECs if lacking proper in situ resolution. Our single-cell analysis showed that PASMCs lack expression of EC markers (Supplemental Figure 4D).

### Transcriptional heterogeneity of PASMCs.

As *ACTA2*^+^ cells have been shown to be major contributors and the origin of SMCs in neomuscularized PAs (8, 9, 11), we focused our further analysis on these cells and investigated their heterogeneity. For this, SMC clusters were subclustered and analyzed in more detail (Figure 4F). Closer examination of cluster distribution within each individual sample highlighted 1 cluster (cluster 4) to be found in only 1 donor sample (Supplemental Figure 4E). To prevent this cluster from overly influencing future cluster-cluster comparisons, we excluded it from further analysis, enabling us to focus on clusters that were reproducibly present in multiple samples. Commonly used k nearest neighbors graph-based clustering uncovered 4 distinct SMC populations in our data set (Figure 4F); however, hierarchical clustering of the top 20 genes per cluster revealed 3 distinct transcriptional profiles, with SMC2 and SMC4 cells being very similar to each other (Figure 4G). To decipher fine, underlying differences between these 2 clusters, we performed pairwise analysis of SMC2 and SMC4 and hierarchical clustering of the top 40 genes. This analysis supported the separation of SMC2 and SMC4 into 2 distinct clusters (Supplemental Figure 4, F and G) and was in line with pseudotime and RNA velocity analysis (Figure 3, D–H) that indicated their diverse origins.

Subsequently, we raised the question whether distinct gene expression profiles for each cluster are associated with a particular set of biological functions. Cluster-enriched genes were subjected to Gene Ontology (GO) biological process analysis evidencing that each cluster possessed a characteristic biological process profile (Figure 4H). SMC1 expressed several genes of the contractile machinery (e,g., *RGS5*, *MYL9*, *ACTG2*), SMC2 was associated with organization of ECM (e.g., *VCAN*, *COL6A3*, *SULF1*) and cellular protein metabolism, SMC3 was enriched in processes for the cellular response to heavy metal ions and regulation of growth (e.g., *MT2A*, *MT1E*, *PHLDA2*, *KLF2*, *GADD45B*), and SMC4 was enriched in complement activation and inflammatory response regulation (e.g., *CD74* and *TMSB4X*) (Figure 4H). We accordingly termed clusters as contractile, synthetic, oxygen sensing, and fibroblast-like. The expression pattern of the top 5 enriched genes (Supplemental Table 5) in each cluster was plotted, showing that contractile cells were defined by prominent expression of *ACTG2*, *CNN1*, *RAMP1*, *RGS5*, and *TPM2*; synthetic cells by *APOE*, *FBLN1*, *LUM*, *TIMP1*, and *VCAN*; oxygen sensing by *FABP4*, *MT1M*, *MT2A*, *RGS16*, and *SOCS3*; and fibroblast-like by *APOD*, *CFD*, *DCN*, *LUM*, and *S100A10* (Figure 4I).

**Figure 5:**
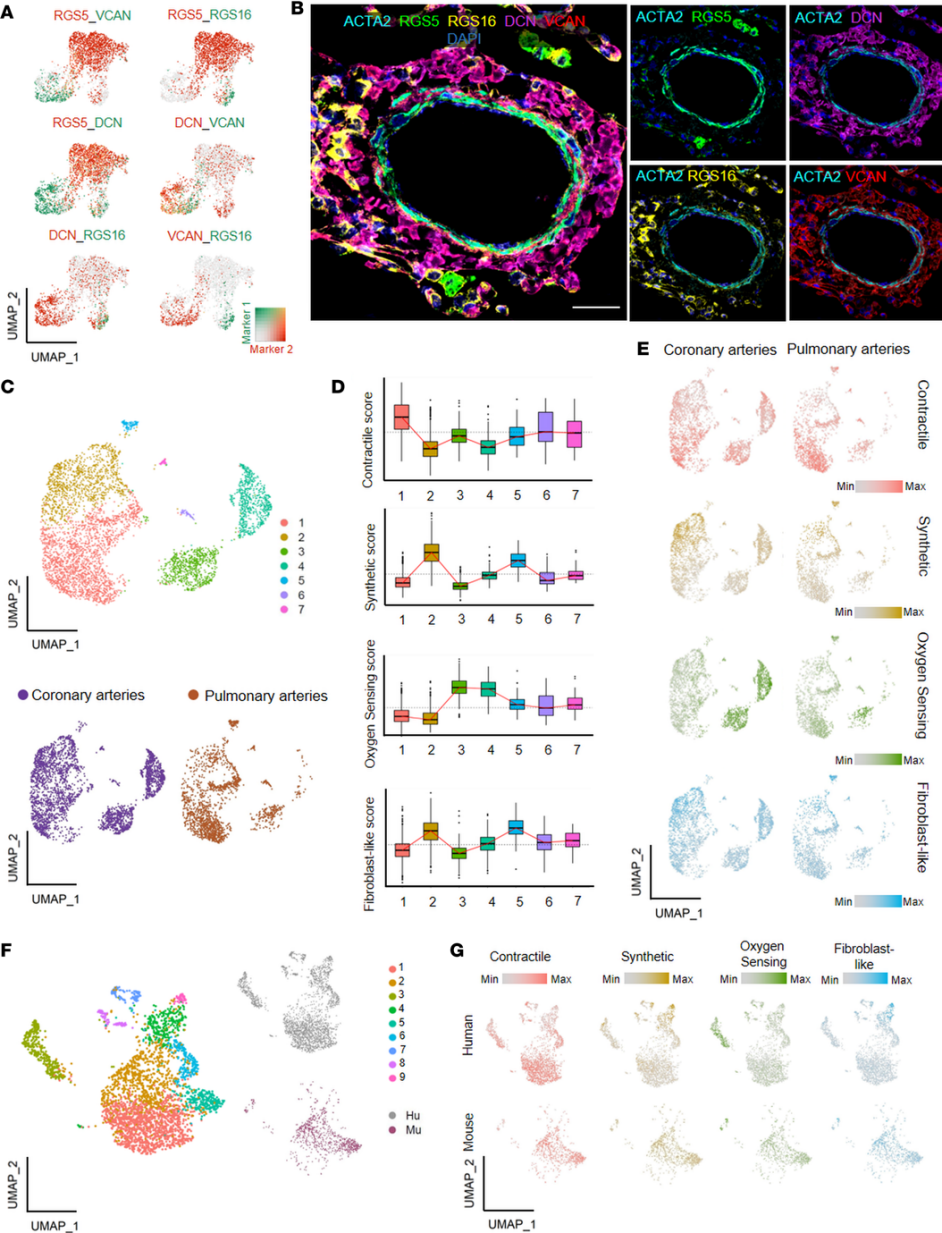
Pulmonary artery smooth muscle heterogeneity is shared between different human vascular beds but not conserved in murine pulmonary artery. (**A**) Uniform manifold approximation and projection (UMAP) expression plots of markers *RGS5*, *DCN*, *RGS16*, and *VCAN* in the 4 smooth muscle cell (SMC) clusters. The color gradient for each marker 1 and marker 2 represents the average expression across the human pulmonary artery (PA) data set. (**B**) Representative immunofluorescence staining of the expression of different SMC population-designated markers in the medial layer of PAs (*ACTA2* in cyan, *RGS5* in green, *RGS16* in yellow, *DCN* in magenta, *VCAN* in red, and DAPI as nuclear counterstain in blue). Scale bar = 50 μm (*n* = 5). (**C**) UMAP of the integration of the human PA single-cell RNA-sequencing (scRNA-Seq) data set and the human coronary artery (CA) scRNA-Seq data set. (**D**) Box plot representing the SMC cluster score in the 7 identified populations of the PA and CA integrated scRNA-Seq data set. SMC cluster scoring was inferred by calculating the average expression of the subpopulation-specific gene query (top 100 cluster-specific genes) and then subtracting the average expression of an equivalent set of randomly selected control genes across the data set. (**E**) UMAP expression plot of the SMC cluster scores in each of the data sets included in the PA and CA integrated scRNA-Seq data set. The color gradient for each score represents the average expression across the entire PA-CA data set. (**F**) UMAP of the integration of the human PA scRNA-Seq data set and the murine PA scRNA-Seq data set. (**G**) UMAP expression plot of the SMC cluster scores in each of the data sets included in the human and murine PA scRNA-Seq data set. The color gradient for each score represents the average expression across the entire human-murine PA data set.

To further investigate the distinctness of the newly identified SMC subclusters, different cluster-enriched genes underwent UMAP overlay. A limited overlap of *RGS5*, *RGS16*, *DCN*, and *VCAN* suggested these as potentially good cluster-designating markers (Figure 5A). To validate these bioinformatic findings, we used high-resolution multi-fluorescence imaging to localize the expression of selected markers of PASMC clusters within the PA in situ (Figure 5B). Although most of the markers were not exclusively expressed by PASMCs (with *DCN* having prominent fibroblast and *RGS5* immune cell expression), all cluster-enriched markers could be observed in the medial layer of PAs together with *ACTA2*.

We next asked about the extent of similarities in vascular SMC composition between different vascular compartments. We took advantage of a publicly available scRNA-Seq data set from human coronary arteries (CAs) (24) and compared the SMC heterogeneity in human PAs and CAs (Supplemental Figure 5A). The integration and overlay of the 2 data sets gave 7 major clusters (Figure 5C). Cells belonging to PASMC and coronary artery smooth muscle cell (CASMC) clusters were homogeneously distributed in the integrated data set, pointing toward preserved cluster designation in vascular beds (Figure 5C and Supplemental Figure 5, A and B). We then inferred specific PASMC cluster scores by taking the top 100 subpopulation-specific markers and overlaid the score in the integrated CASMC and PASMC data set. We observed the preservation of contractile, synthetic, and fibroblast-like populations; however, CASMCs possessed an additional oxygen sensing population. PASMC synthetic and fibroblast-like populations closely resembled each other (Figure 5, D and E; and Supplemental Figure 5, C and D).

Additional analysis investigated the extent of interspecies similarities within PASMC populations. Integration of human and murine *ACTA2*-expressing cells showed that murine PASMCs were characterized by lower level of heterogeneity compared with humans (Figure 5F). Similar to the PA-CA data set, we inferred the same specific PASMC cluster scores and overlaid the cluster on the integrated human-murine PA data set. This revealed a prominent signature of contractile and oxygen sensing populations in the murine data set. However, the synthetic and fibroblast population pattern was spread among all murine *ACTA2*-expressing cells, without defined clusters (Figure 5G and Supplemental Figure 5E).

### PASMC phenotypic shift upon pulmonary vascular remodeling.

**Figure 6:**
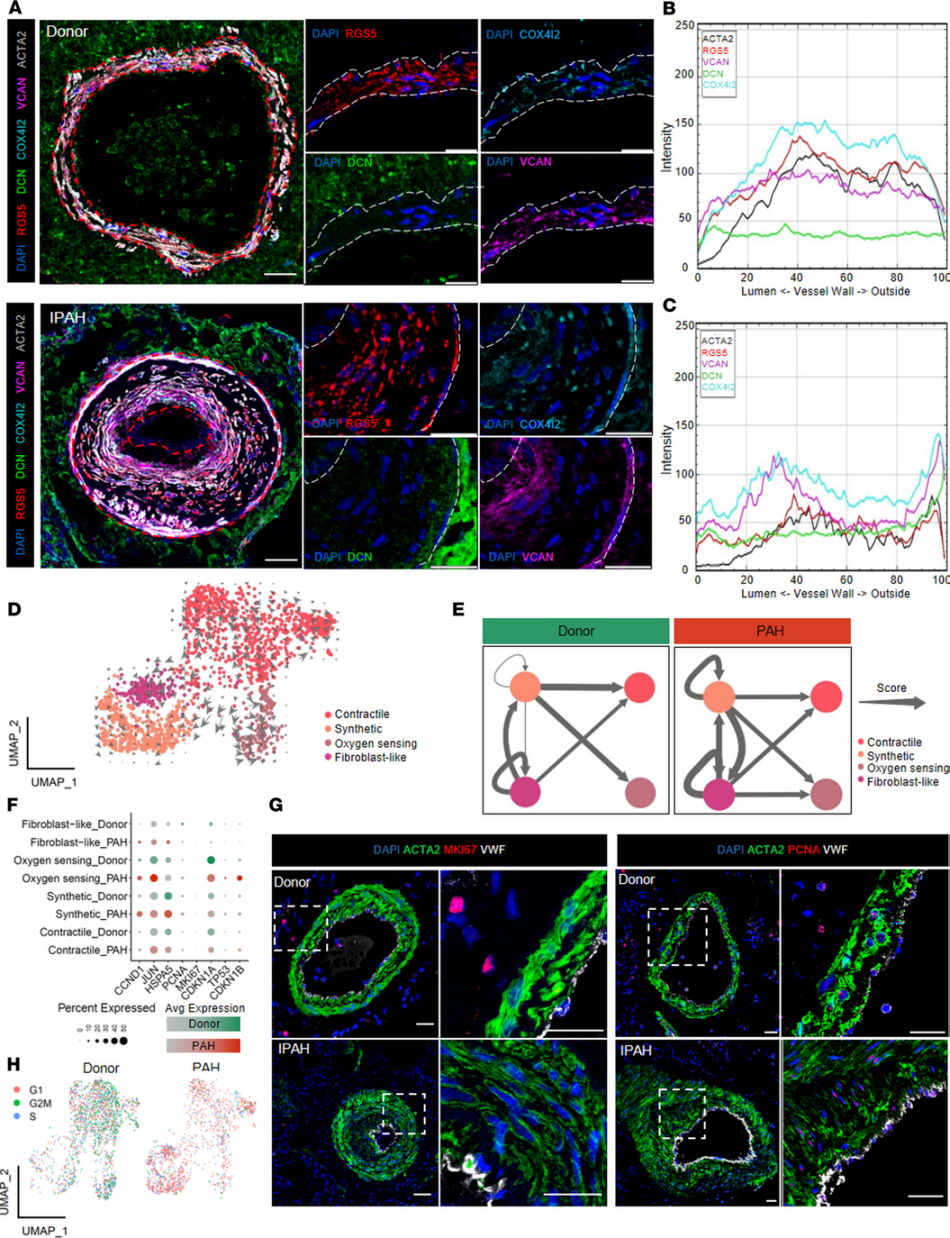
Vascular remodeling causes phenotypic shift among pulmonary artery smooth muscle cell clusters. (**A**) Representative immunofluorescence staining of 4 smooth muscle cell (SMC) clusters in donors (*n* = 4; pulmonary artery, PA, *n* = 7) and idiopathic pulmonary arterial hypertension (IPAH, *n* = 5; PA, *n* = 7) patients (*RGS5* in red, *DCN* in green, *COX4I2* in cyan, *VCAN* in magenta, *ACTA2* in gray, DAPI in blue). Scale bar = 20 μm. Representative intensity (mean fluorescence intensity [arbitrary units]) histograms of the distribution of SMC cluster markers in donor (**B**) and IPAH (**C**) PA. (**D**) RNA velocity calculation overlaid on uniform manifold approximation and projection (UMAP) of the SMC subset. RNA velocity analysis identified 219 velocity genes across the data set. (**E**) Ligand-receptor analysis between SMC clusters in donor and PAH PAs. Permutation test (*n* = 100,000), *P* < 0.05. Arrows’ thickness is relative to total number of interaction pairs. (**F**) Dot plot of 8 proliferation-related genes. Dot size represents percentage of cells expressing the gene; color gradient represents the average expression across the SMC data set. (**G**) Representative immunofluorescence staining of *MKI67* (*n* = 3 donors/5 PA; *n* = 5 IPAH/16 PA) and *PCNA* (*n* = 4 donors or IPAH, 21 PAs from donors or IPAH) in PA from donor and IPAH tissue samples (*ACTA2* in green, *MKI67* or *PCNA* in red, *VWF* in gray, and DAPI in blue). Scale bar = 20 μm. (**H**) UMAP of cell cycle scoring for G1, G2M, and S phase in the extracted SMC data set from donor and PAH PAs.

We next set out to identify the localization of the 4 SMC clusters (Supplemental Figure 6A) in normal and remodeled PAs using confocal imaging of multicolor-stained lung tissue slides. In situ localization analysis showed quite homogeneous distribution of the SMC subpopulation markers throughout the PA medial layer in healthy conditions (Figure 6, A and B, and Supplemental Figure 6B). *DCN*-expressing cells were mainly localized on the outer regions of the vascular wall, and only a limited fraction of *DCN*-expressing SMCs (fibroblast-like SMC) were found in the medial layer, as attested by the dim intensity of *DCN* fluorescence signal. The fluorescence intensity profile of *RGS5* (contractile SMC), *VCAN* (synthetic SMC), and *COX4I2* (oxygen sensing SMC) suggested that these subpopulations are spread evenly throughout the medial layer. Pulmonary vascular remodeling displayed a noticeable shift in SMC cluster localization. The most prominent feature was frequent presence of *VCAN*-positive and *ACTA2-*dim SMC in the neointimal region (Figure 6, A and C, and Supplemental Figure 6B). Weaker expression of *ACTA2* in neointimal cells is indicative of SMC phenotypic shift defined by partial loss of mature contractile markers and production of ECM proteins. UMAP biplots of contractile marker *DES* or the synthetic one *COL1A1* coexpression with *VCAN* showed clear distinction between contractile and synthetic clusters in donor and PAH samples with predominant *COL1A1* expression in noncontractile SMCs (Supplemental Figure 6C). Of note, while fibrillar collagens are traditionally considered a hallmark of the synthetic SMCs (25), the high expression of the proteoglycan class of ECM genes (*VCAN*, *BGN*, *DCN*) seems to be defining feature of synthetic and fibroblast-like clusters (Supplemental Figure 6D).

In the next step, we investigated if there is a redistribution of SMCs among identified clusters in PAH compared with donor samples, since there was no indication of cluster loss or gain in PAH samples. Trajectory inference and RNA velocity analysis indicated a shift from central contractile cluster into 2 main directions: one branch leading to synthetic and fibroblast-like cluster cells and the second toward oxygen sensing (Figure 6D and Supplemental Figure 6, E and F). Further support of inferred dynamic shift was a general redistribution in the relative proportions of SMC clusters, with elevated fraction of synthetic SMCs and decreased contractile SMCs in PAH compared with donor samples (Supplemental Figure 6G). Cellular redistribution was also accompanied by a shift in signaling interactions among SMC clusters (calculated as weighted ligand-receptor pairs), with PAH showing a denser signaling network originating in synthetic SMCs (Figure 6E).

This prompted us to focus on potential functional differences among SMC clusters between normal and diseased states. Proliferation and thus expansion of SMCs is postulated as a key pathogenic mechanism of pulmonary vascular remodeling. Analysis of common positive (*CCND1*, *JUN*, *HSPA5*, *PCNA*, *MKI67*) and negative (*CDKN1A*, *CDKN1B*, and *TP53*) regulators of cell cycle progression showed a mixed picture with generally increased expression of both gene sets in PAH (Figure 6F). Similar results were also reproducible in the murine setting (Supplemental Figure 6H). Staining with a diagnostically used antibody against *MKI67* revealed very rare SMC staining in both conditions (Figure 6G and Supplemental Figure 6I). Staining with an alternative marker, *PCNA*, resulted in a broader staining pattern compared with *MKI67* (Figure 6G and Supplemental Figure 6I) explainable by the involvement of PCNA in biological processes other than proliferation. Image analysis in both cases indicated lower percentage of proliferation marker–positive SMCs in PAH compared with donor PAs (Supplemental Figure 6I). To obtain a conclusive result on the proliferation state of PASMC clusters using a less biased approach, we performed a cell cycle phase scoring analysis based on a larger set of proliferation-regulated genes. Results of this analysis were in line with in situ proliferation marker stainings and indicated a skewed ratio of all SMC clusters toward G1 cell cycle phase in PAH compared with donor (Figure 6H and Supplemental Figure 6J). Similar analysis done on murine SMCs showed a remarkably similar pattern of SMCs isolated from chronic hypoxia PAs, with the majority of cells being in G1 phase (Supplemental Figure 6K).

### Pulmonary vascular remodeling is associated with cluster-specific transcriptome response.

**Figure 7:**
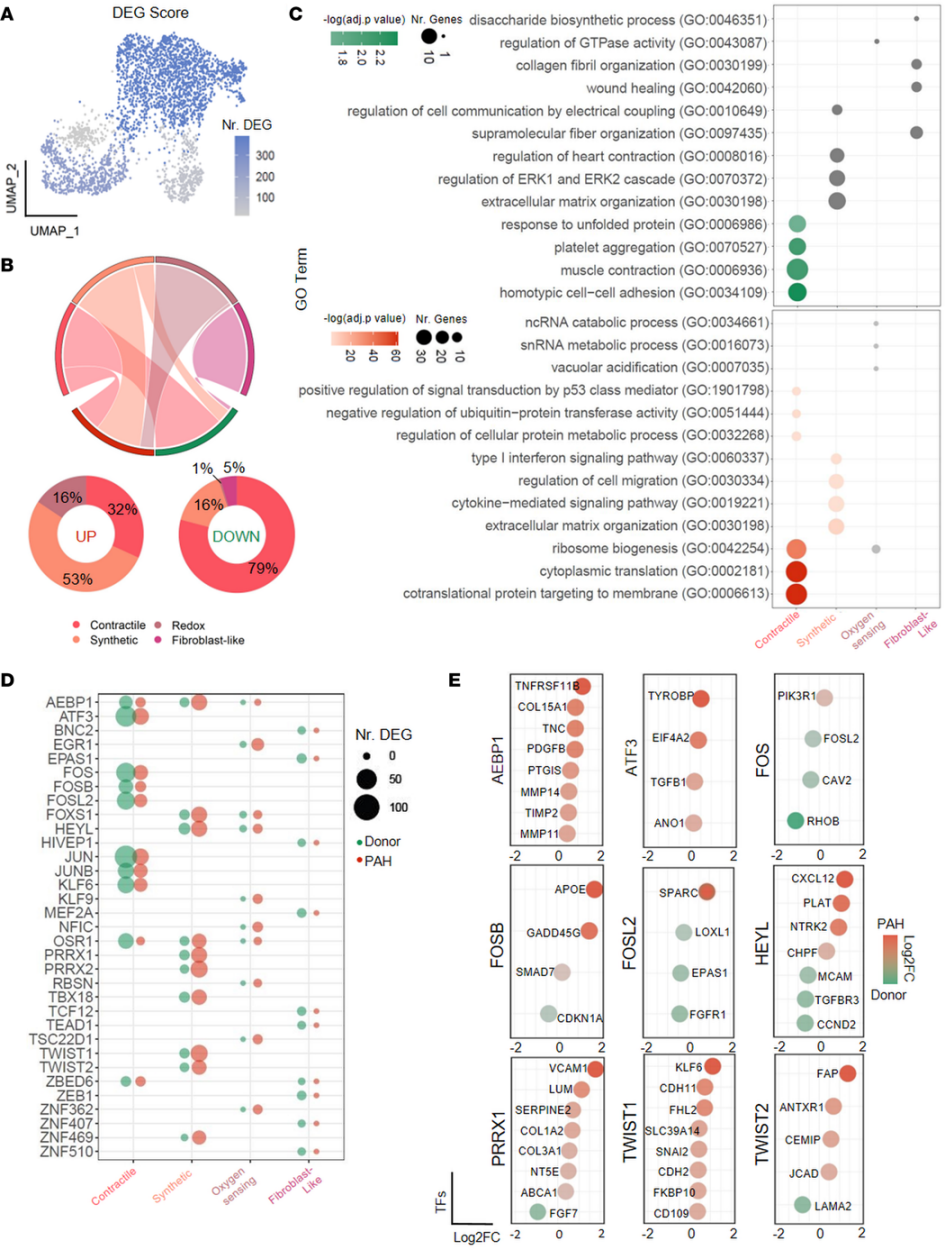
Smooth muscle cells feature cluster-distinct regulation of gene expression upon pulmonary vascular remodeling. (**A**) Uniform manifold approximation and projection (UMAP) with overlay counting of the differentially expressed genes (DEGs) in healthy and remodeled pulmonary arteries (PAs) in each smooth muscle cell (SMC) cluster. Wilcoxon rank sum test with Bonferroni adjustment, *P* < 0.05 and |log_2_(fold change)| > 0.25. (**B**) Chord diagram and associated pie charts of DEGs upon vascular remodeling in each SMC cluster. (**C**) Gene ontology (GO) enrichment analysis resulting from down- and upregulated DEGs upon vascular remodeling in each SMC cluster. Dot size depicts number of genes included in each specific GO term; color-coding corresponds to significance. Fisher’s exact test with Benjamini-Hochberg adjustment, *P* < 0.05. (**D**) Dot plot representing DEGs enriched in specific transcription factors in donor (green) and PAH (red) from ChEA3. (**E**) Dot plot depicting regulation (log_2_ fold change) for several manually selected genes in contractile and synthetic SMC clusters and their putative transcription factor regulator.

We next investigated remodeling-associated gene expression changes in a cluster-specific manner. DEG analysis between donor and PAH cells displayed very limited overlap in the significantly regulated genes, with PAH cells possessing larger numbers of uniquely regulated genes (Figure 7A and Supplemental Table 6). The smallest numbers of DEGs were present in fibroblast-like and oxygen sensing clusters, while synthetic and contractile PASMCs showed the strongest transcriptionally distinct signature in PAH (Figure 7A). Interestingly, the contractile cluster was associated primarily with downregulation and synthetic with upregulation of the gene transcription (Figure 7B). Pathway enrichment analysis based on GO biological process showed that downregulated genes in the contractile cluster were involved in muscle contraction and cell adhesion pathways, while upregulated ones were responsible for ribosomal biogenesis and translation processes (Figure 7C). The synthetic cluster displayed downregulation of ERK signaling cascade and heart contraction pathway and upregulation in type I interferon signaling as well as ECM organization processes and cell migration in PAH (Figure 7C). Several of the upregulated genes in the synthetic cluster have previously been associated with PAH pathogenesis, such as *CXCL12*, *TNFRSF11B* (osteoprotegerin), and *PDGFD* (26–29) (Supplemental Table 6). A distinct pattern of gene expression changes pointed to a potential cluster-specific regulatory network, and analysis of transcription factor enrichment on DEGs indeed supported that genes in each cluster are regulated by different transcription factors (Figure 7, D and E, and Supplemental Table 7). The regulatory hub in the contractile cluster was centered on AP-1 family members and *ATF3*, both involved in mediating cell stress responses (Figure 7, D and E). In contrast, the synthetic cluster network contained transcription factors involved in differentiation processes such as *TWIST1* and *TWIST2*, regulator of NOTCH signaling *HEYL*, and transcriptional regulator of fibrillar collagen expression *AEBP1* (Figure 7, D and E). In summary, each PASMC cluster showed specific gene expression changes upon remodeling, pointing to cluster-wise functionality and dynamic shifts upon vascular remodeling.

## Discussion

Pulmonary vascular disease affects up to 70 million people worldwide (30) and directly contributes to increased patient mortality and decreased quality of life. Expansion of *ACTA2*-expressing cells is one defining feature of pulmonary vascular disease, resulting in vascular wall thickening and lumen reduction (9, 31). So far, analysis of the gene expression changes underlying vascular remodeling has primarily been restricted to the analysis of total lung tissue or of laser-microdissected vessels (5, 32). While the former is limited by sampling bias, as shown by the underrepresentation of vascular cells (33–35), the latter is extremely laborious and time-consuming. Single-cell transcriptomics permits an unbiased, marker-agnostic approach to investigate tissue cellular composition at an unprecedented resolution. Using this single-cell approach, we provide overall insight into the global cellular changes occurring in pulmonary vascular disease and detailed analysis of PASMC heterogeneity and underlying transcriptional behavior during vascular remodeling.

Although an altered composition of both structural and immune cell populations in the diseased PA is well documented (7, 9, 11) and further supported by the current study, the significant presence of immune cells in normal, healthy PAs is often overlooked. Using multicolor immunofluorescence staining of thick lung tissue pieces and 3D rendering of confocal images, we localized immune cells in the perivascular region and the vessel wall of normal donor PA. These immune cells are an integral part of the normal PA niche, and their presence and balanced composition are likely a part of vascular homeostasis. This is further supported by our finding of extensive cellular communication based on ligand-receptor expression between different immune cell populations and vascular structural cells. Moreover, our current study unravels a striking shift in signaling interactions and breakdown in communication between structural and immune cells in PAH. Signaling in PAH centers predominantly on structural cells. PASMCs in particular increase both receptor and ligand expression. This could imply that PAH PASMCs extend their function beyond being a signal responder cell population and gain an active role in directing the remodeling process. Supporting this notion is gene expression analysis showing the PASMC synthetic cluster having enriched expression of soluble factors, such as *IL-8*, *CXCL12*, *TNFRSF11B*, and *PDGFD*, which are associated with PAH pathogenesis (26–29).

PASMCs, as central players in pulmonary vascular remodeling, thus have both structural and functional roles. Studies investigating bovine SMCs have shown that PASMCs have roles beyond simple contractile response and display differences in morphological appearance, marker expression, and proliferative capacity (36). Studies on murine PASMCs, using a lineage tracing approach, have indicated that a specific PASMC subpopulation is responsible for neomuscularization (14, 37). We believe our current study is the first one resolving the human PASMC heterogeneity in healthy and remodeled PAs by using an unbiased, marker-agnostic approach. We identified 4 PASMC clusters that showed enrichment in a distinctive set of biological processes. Based on enrichment in contraction, signaling/oxygen sensing, ECM organization, and complement/immune regulation processes, we assigned putative functional roles to each of the 4 clusters and named them as contractile, oxygen sensing, synthetic, and fibroblast-like, respectively.

Synthetic and fibroblast-like clusters possessed a similar transcription profile characterized by shared expression of ECM components and processing enzymes. However, fibroblast-like cluster cells have additional expression of adventitial fibroblast-enriched genes, such as *DCN* and *C3*. Further bioinformatic analysis using trajectory, pseudotime scoring, and RNA velocity implied that synthetic cluster cells are most likely derived from SMCs, while fibroblast-like cells might arise from both fibroblasts and SMCs. The coexpression of *ACTA2* and *DCN* in the fibroblast-like cluster resembles a rare cell population in murine PAs labeled by SMC (*ACTA2*) and fibroblast (*PDGFRA*) lineage markers (9, 11). Nevertheless, our current data demonstrate that transcriptionally distinct programs underly PASMCs and adventitial fibroblasts, which comprise a very homogeneous population that can be defined by a set of classical markers such as *DCN* and *PDGFRA* or potentially novel ones such as *SERPINF1*, *C3*, and *SLPI*. Additionally, contractile and oxygen sensing clusters were also highly related, both having high expression of contraction-related genes. However, the oxygen sensing cluster showed high expression of heavy metal ion binding proteins metallothioneins, the GPCR signaling regulator *RGS16*, and electron transport chain component *COX4I2*. *COX4I2* is indispensable for oxygen sensing (22, 38), while metallothioneins could regulate availability and intracellular zinc levels and thus indirectly influence hypoxia-sensing in PASMCs (39). The presence of this specialized oxygen sensing SMC population is supported by similar findings using scRNA-Seq of murine carotid body oxygen sensing cells (40). Transcriptomic profile of oxygen sensing SMCs was preserved in both murine PA and human CA data and in both cases associated with putative pericyte markers. We could not find a distinct transcriptional signature for pericytes, with the majority of classical pericyte markers, such as *PDGFRB*, *RGS5*, *CSPG4*, also being expressed in other PASMC clusters. A shared transcriptional signature between pericytes and SMC has already been reported in previous studies, and ultrastructure analysis still remains the prerequisite condition for pericyte identification (9, 41). On closer examination, we detected a subgroup of cells in the oxygen sensing cluster with particularly high expression of pericyte markers, such as *RGS5* and *NDUFA4L2*. *NDUFA4L2* is, similar to *COX4I2*, a mitochondrial electron chain protein with a role in development of hypoxia-induced PH (42), indicating that pericytes might be involved in oxygen sensing.

Our results indicate that the redistribution of PASMCs between clusters is characteristic of pulmonary vascular remodeling, rather than loss of existing or appearance of novel populations. This finding was expanded and validated by localization analysis showing enriched presence of synthetic cluster marker *VCAN* in the neointima region. These findings are in line with a previous proteomics-based approach (43), which drew similar conclusions in whole lung tissue — that SMC-dependent vascular remodeling is defined by a shift from contractile toward synthetic phenotypes. A similar phenotypic shift toward synthetic phenotype was also observed in SMCs from remodeled coronary arteries (24), and we show that the transcriptional profile of all 4 PASMC clusters is shared with coronary SMC clusters. In contrast, murine PASMCs showed a lower level of heterogeneity compared with human caused by the very low abundance of synthetic and fibroblast-like PASMCs. This intrinsic difference in human and murine PASMC heterogeneity could in part explain the lack of neointima formation in most of the murine pulmonary vascular disease models. In line with the proposed phenotypic shift, the contractile cluster downregulated genes belonging to muscle contraction, while upregulating genes related to the hypertrophic response. Interestingly, regulated genes in the contractile cluster seemed to be under the transcriptional control of *ATF3* and AP-1 transcription factors — hallmark and mediators of stress responses. In contrast, the synthetic cluster upregulated proinflammatory and growth/differentiation factors, in addition to ECM components. Strikingly, the regulatory network in synthetic cluster encompassed transcription factors associated with (trans)differentiation processes, such as *TWIST1*&*2*. It also included a Notch pathway effector, *HEYL*, supporting previous observations of a central role of Notch3 signaling in PAH pathogenesis (44–46).

Another interesting finding of the current study is the balanced distribution of cluster-designated cells in different cell cycle phases and the enrichment of PAH PASMCs in G1 cell cycle. Skewed cell cycle profile toward G1, and consequently less G2M and S phase, would imply that the cells are in synthetic cell growth and not in an active proliferating phase. This at first seems counterintuitive, as PASMC proliferation is postulated as a key pathological feature of vascular remodeling. However, PASMC proliferative burst is probably an early and transient process, as attested by well documented studies on animal models, and happens prior to beginning of measurable remodeling (8, 9). In established disease, such as the end-stage disease samples used for this study, PASMCs in remodeled vessels rarely contain BrdU-positive/proliferative cells (8).

One remaining open question is how flexible or permanent each PASMC cluster assignment is for a particular cell. In other words, is each identified PASMC cluster representing a predetermined and distinct sublineage or a transient cell state amendable to phenotypic modulation? The presence of different transcriptome profiles and their shifts indicates that pathologic remodeling could potentially be reversed by therapies that regulate phenotypic behavior and direct the PASMC cell state decision toward a homeostatic, healthy profile.

In summary, the current study through its compartment-specific approach complements current efforts to establish a lung cellular atlas and provides a blueprint of molecular changes associated with pulmonary vascular remodeling at single-cell level. It identifies a breakdown in normal intercellular communication, a heterogenous PASMC population, and skewed cellular distribution between distinct PASMC clusters in pulmonary vascular disease.

## Methods

### Human lungs.

Human lung samples (Supplemental Table 8) were collected at Department of Thoracic Surgery, Medical University of Vienna, Vienna, Austria (lungs from patients listed for having IPAH and downsized donor lungs) and University of Pennsylvania, Philadelphia, Pennsylvania, USA (nonutilized donor lungs).

### PA isolation.

PAs were identified in lung tissue slices based on close proximity to airways. Only medium to small (diameter < 5 mm) PAs were dissected under a stereomicroscope (SZX7, Olympus) by following the branching pattern and including accompanying perivascular (adventitial) region. Arteries were minced and digested for 60 minutes at 37°C in phosphate-buffered saline (PBS) containing 2 mg/mL collagenase A (Roche) and DNase I (MilliporeSigma). Digest was filtered through 100 μm cell strainer into FBS and diluted with PBS. Following centrifugation (400*g*, room temperature, 5 minutes), cell pellet was washed with PBS and passed through 40 μm cell strainer (Falcon, Corning). When necessary, an erythrocyte lysis step was performed in ammonium chloride/potassium bicarbonate/EDTA (MilliporeSigma) buffer for 2 minutes at room temperature. Cell pellet was resuspended in PBS containing 0.03% bovine serum albumin and used for scRNA-Seq capture or in magnetic activated cell sorting (PBS, 2 mM EDTA, 0.5% bovine serum albumin) buffer for flow cytometric analysis. The secondary and tertiary PAs from the left lung lobe were isolated from the lungs of 12-week-old male mice [wild-type littermates of B6(Cg)-(TgActa2-cre/ERT2)12Pcn mice, MGI ID 3831907, sourced from P Chambon and D Metzger, Institut de Génétique et de Biologie Moléculaire et Cellulaire, Illkirch, France, crossed with B6.Cg-*Gt(ROSA)26Sor^tm14(CAG-tdTomato)Hze^*/J mice, strain 007914, sourced from The Jackson Laboratory] kept under normobaric hypoxia (10% O_2_) or normoxia (21% O_2_) for 3 weeks and minced as described above. Development of PH was indirectly confirmed by increased Fulton index (weight ratio of right ventricle to left ventricle and septum). Hypoxia exposure and organ collection were approved by the institutional review board (University of Pennsylvania, Philadelphia, Pennsylvania, USA). Isolated PAs were processed the same way as human PAs involving mechanical and enzymatic processing. Single-cell suspensions of PAs from 3 mice per condition were pooled together and used for 10x Genomics Chromium capture and Gel bead-in-Emulsion generation.

### 3D immunofluorescence staining.

Fresh human lungs were inflated with 2% low-melting-point agarose (MilliporeSigma); 200 μm thick, precision-cut human lung tissue sections were cut using a tissue slicer (Krumdiek), fixed overnight in methanol-DMSO (4:1), washed in PBS, and permeabilized overnight (5% bovine serum albumin, 0.5% Triton X-100 in PBS). Incubation with primary and secondary antibodies (Supplemental Table 9) was done in 0.3% Triton X-100 at 4°C. In case of TSA-based detection, Immpress HRP Polymer (Vector Laboratories) was used in combination with AlexaFluor TSA dyes (Thermo Fisher Scientific).

### 3D rendering.

3D image rendering was performed in Imaris 9.8 (Bitplane). After performing surface rendering of the *ACTA2* channel, identification of the total immune cells was performed by first spot rendering the *CD45* channel and second selecting cells with a diameter of approximately 7 μm. Intercalated immune cells were then highlighted using the “find spot inside surface” incorporated plugin for generating intercalated spots (corresponding to immune cells) in the *ACTA2* surface.

### scRNA-Seq and data analysis.

The pulmonary arterial single-cell suspension was captured for barcoding and RNA sequencing using Chromium instrument (10x Genomics), and cDNA libraries were prepared using 10x Genomics Kit V2 according to manufacturer’s instruction and purified using SPRIselect beads (Beckman Coulter). Quantification and quality control of prepared libraries were done using Bioanalyzer (Agilent). Libraries were sequenced at the Biomedical Sequencing Facility at CeMM using Illumina HiSeq4000 with single-pair 75 bp reads. Preprocessing the scRNA data was performed with Cell Ranger v3.0.0 (10x Genomics). Raw sequencing files were demultiplexed and aligned against the human reference genome GRCh38. More detailed analysis was performed in Seurat (version 3.2) (47) in the R environment (version 3.6). Each sample was individually processed and then integrated using Seurat computed anchor set. Quality control filtered out cells with >5% mitochondrial genes and either very low (<200) or very high (3 times the median absolute deviation of genes) number of genes. Variable genes were identified with the Seurat *FindVariableFeatures*, using default parameters. The Seurat *ScaleData* function was used to scale and center expression values and remove potentially unwanted sources of variation (UMI count data, percentage mitochondrial and cell cycle scoring). Principal component (PC) analysis for dimensional reduction was performed with Seurat functions based on the variable genes previously identified. Shared nearest neighbor graph was performed with the first 20 PCs and clustered using the Louvain method with a resolution of 0.2. Further dimension reduction was performed using UMAP for dimensions reduction algorithm or t-distributed stochastic neighbor embedding using the first 20 PCs. DEG analysis was done on an integrated RNA slot in the case of SMC cluster markers. Conserved genes within each cluster were identified using *FindConservedMarkers* for each cluster and used to manually annotate each cluster. Marker genes for each cluster were statistically identified with the *FindAllMarkers* method using MAST (48) and log-fold change threshold of 0.25 and expressed in a minimum of 25% of the cluster. Differential gene expression between donors and PAH clusters was identified using the *FindMarkers* method using standard parameters. The processed data were then further analyzed for gene-gene interactions and enriched GO term annotations (GO biological processes) using the top 30 entities from each cluster. The interaction network was created with the stringApp for Cytoscape using the default query settings without adding additional interacters (49). Subsequent functional enrichment analysis for GO biological processes (50) was performed separately for each cluster and visualized by Cytoscape using a redundancy cutoff of 0.5 (48). Ligand-receptor interaction analysis was performed using *scTalk* according to developers’ guidelines (49), taking advantage of a human ligand-receptor pairs database, adding weights after reference to the STRING database. The final interaction weight for each ligand-receptor pair corresponded to the sum of the weights from the expression of the given ligand in the source cell (determined by log_2_ fold change), the interaction weight between ligand and receptor retrieved from STRING database, and the weights from the expression of the given receptor in the target cell (determined by log_2_ fold change).

Integration of human PA and human CA scRNA-Seq data was performed following the same integration guidelines as mentioned before, whereas the cross-species integration between human PA and murine PA scRNA-Seq data was preceded by conversion of the official murine gene symbol nomenclature in the human one to avoid batch effect during the integration procedure.

### Trajectory inference and pseudotime calculation.

Trajectory inference and pseudotime calculation were computed using Monocle 3 according to developers’ guidelines (50–52). Briefly, preprocessing steps such as variable feature identification, normalization, scaling, dimensionality reduction, and clustering performed in Seurat were used as input for the *learn_graph* command in Monocle 3. Nodes belonging to fibroblast and SMC clusters were used as origin nodes for pseudotime calculation in 2 independent runs using the command *order_cells*. The single cell–specific pseudotime values were then extracted using the pseudotime function in Monocle 3 and attached in the metadata of the original Seurat file for further visualization.

### RNA velocity calculation.

RNA velocity was computed using scvelo version 0.2.4 according to developers’ guidelines (53). Briefly, loom files for each 10x Genomics run were generated using velocyto (54) and then filtered according to cells in the fibroblasts and SMC or SMC data subsets, respectively. For each gene, RNA velocity was computed fitting a dynamical modeling. First, genes that did not fulfil the default parameter of coefficient of determination (velocity_r2 < 0.01) and degradation rates (velocity_gamma < 0.01) were filtered out. Second, to address the dynamics behind the remaining velocity genes, a dynamic modeling was fitted to infer transcription rates, splicing rates, and degradation rates. Finally, genes with a fit_likelihood<=min_likelihood of 0.001 were filtered out. This allowed us to identify 317 velocity genes in the fibroblast and SMC data subset and 219 velocity genes in the SMC data subset that were used to infer single-cell dynamics in the data set.

### Immunofluorescence staining.

Formalin-fixed, paraffin-embedded lung tissue sections (2.5 μm) were deparaffinized and rehydrated in decreasing concentrations of ethanol. Antigen retrieval was performed in sodium citrate pH 6 buffer. Blocking was performed using 5% donkey serum, followed by another blocking step in 3% bovine serum albumin in PBS. Sections were incubated with primary antibodies (Supplemental Table 9) overnight at 4°C, followed by washing and incubation with Alexa Fluor 555/647–labeled secondary antibodies (Life Technologies) at room temperature for 2 hours. Negative controls were performed in parallel by omission of the first antibody. We used 20× plan apochromatic multi-immersion objective (0.75 numerical aperture, Leica) and apochromatic glycerol immersion objectives 40× (1.25 numerical aperture, Leica) for image acquisition.

### Multicolor fluorescence imaging.

Formalin-fixed, paraffin-embedded lung sections were dewaxed and subjected to heat-induced antigen retrieval (pH 6). Multiplex immunofluorescence staining was done using Opal kit (Akoya). Successive rounds of primary antibody (Supplemental Table 9), detection reagent (Opal Polymer HRP, Akoya; or Immpress HRP Polymer, Vector Laboratories), and fluorescence signal development (Opal dyes, Akoya or AlexaFluor TSA, Thermo Fisher Scientific), followed by antibody removal, were performed according to manufacturer’s instructions. DAPI (Thermo Fisher Scientific) was used as a nuclear counterstain at 2.5 μg/mL final concentration. Slides were imaged using SP8 fluorescence confocal microscope (Leica) and apochromatic glycerol immersion objectives 40× (1.25 numerical aperture) and 63× (1.30 NA). Acquired image data were processed using Lightning detection package (Leica) for adaptive image reconstruction using default settings. Spectral overlap was compensated using Channel Dye Separator in manual mode (Leica).

### SMC subpopulation immunofluorescence localization assessment.

Automated image analysis was performed in FIJI (ImageJ v 1.53q) (55). Initially the full vessel shape was assessed via combining the maximum intensity projections of channels containing *COX4I2*, *ACTA2*, *RGS5*, and *VCAN* information, generating regions of interest (ROIs) for the vessel lumen and the outer circumference. The center of mass of the lumen was calculated and used as the point of origin for 32 evenly spaced measuring probes. These probes were designed to automatically detect the fluorescence intensities, rescale them to a common baseline to represent the vessel wall composition from lumen toward the outside, and average the measurements of all 32 probes. Graphical output represents the mean of these 32 probes with saturation set to 0.35 for ease of viewing.

### KI67 and PCNA immunofluorescence quantification.

Quantification was performed by developing a Fiji-based (ImageJ v 1.53q, NIH) macro. Following an initial maximum intensity projection, channels containing either *MKI67* or *PCNA* were thresholded based on Yen’s automated multilevel thresholding method (56). Subsequent nucleus detection and water shedding were performed, and individual nuclei were determined to be part of the medial layer if the dilated ACTA2 signal was found to be overlapping without VWF signal being present within the nuclear region. Masks generated of nuclei positively identified to belong to the medial layer were used as ROIs in the *MKI67* or *PCNA* channel, respectively, and output is given as percentage of positive signal based on nucleus area. We provide the in-house-developed tool for the analysis under the name of “Proliferation Marker Expression” macro on Github (https://github.com/JGHMicroscopy/Fiji-Macros/blob/c2bc729547b3580b0f7d824af25f3a2e979d9815/Proliferation%20Marker%20Expression.ijm; commit ID c2bc729).

### Transcription factor analysis.

Transcription factor (TF) analysis was performed using ChEA3 (57). Disease-associated, cluster-specific DEG terms were provided as gene queries in 4 independent enrichment runs. Results were ranked based on how consistently the given TFs were found connected to the given gene query in different data libraries, such as ChIP-Seq databases (ENCODE, ReMap), coexpression of TFs with other genes based on processed RNA-Seq data from GTEx and ARCHS4 and, finally, co-occurrence of TFs with other genes by examining gene lists submitted to the tool Enrichr (38, 58, 59).

### Data availability.

Results for human and mouse scRNA-Seq were uploaded to the National Center of Biotechnology Information Gene Expression Omnibus database (accession number GSE210248).

### Statistics.

Wilcoxon rank sum test was used to identify cluster-specific DEGs via the Seurat package (version 3.6) using the function *FindAllMarkers* with Bonferroni correction and a threshold of adjusted *P* value of 0.05 and log_2_ fold change of ±0.25. Comparison of DEGs between donor and PAH samples was performed using the *FindMarkers* function and Wilcoxon rank sum test with Bonferroni correction and a threshold of adjusted *P* value of 0.05 and log_2_ fold change of ±0.25. Cell proportions and SMC populations’ proportions statistical differences between donor and PAH were inferred with the propeller function of the speckle R package. Arcsine transformation was performed prior to assessing statistical significance through 2-tailed *t* test in each cell type or SMC population, with a *P* value threshold of 0.05. Significant ligand-receptor interactions were highlighted through permutation test with number of permutations set on 100,000, as suggested by developers. Statistical analysis of significant differences between donor and IPAH staining of *MKI67*/*PCNA* were addressed through Wilcoxon rank sum test with a *P* value threshold of 0.05. Disease-associated DEG analysis between the 4 clusters of SMCs was performed using *FindAllMarkers* function and Wilcoxon rank sum test with Bonferroni correction and a threshold of adjusted *P* value of 0.05. Enrichment analysis was performed on Enrichr using Fisher’s exact test followed by Benjamini-Hochberg correction with an adjusted *P* value threshold of 0.05.

### Study approval.

Collection of human tissue and clinical data was done in accordance with the Declaration of Helsinki and following patient written informed consent and protocol approval by local institutional review boards (Medical University of Vienna, Vienna, Austria, 976/2010; University of Pennsylvania, Philadelphia, Pennsylvania, USA, PROPEL). Hypoxia exposure and murine organ collection were approved by the institutional review board (University of Pennsylvania, Philadelphia, Pennsylvania, USA, 806345).

## Author contributions

SC, LMM, and GK designed and planned the study. JK, EG, WK, EC, and JL provided the human material. SC, FV, EF, JG, MB, and HW were involved in data acquisition. FV, JG, HTP, MPM, and LMM performed data analysis. SC, FV, HO, YYZ, EEM, LMM, and GK interpreted the data. SC, FV, EF, LMM, and GK drafted the manuscript. SC and FV share the first author position because both authors were critically involved in data acquisition and interpretation underlying all figures, as well as manuscript preparation. The authorship order between them was assigned because SC designed the study and performed underlying experiments.

## Supplementary Material

Supplemental data

Supplemental table 1

Supplemental table 2

Supplemental table 3

Supplemental table 4

Supplemental table 5

Supplemental table 6

Supplemental table 7

Supplemental table 8

Supplemental table 9

## Version 1 09/13/2022

In-Press Preview

## Version 2 10/24/2022

Electronic publication
