# Supplementary material for: Single-cell transcriptomics reveals skewed cellular communication and phenotypic shift in pulmonary artery remodeling
Source: JCI Insight. 2022 Oct 24;7(20):e153471. doi: 10.1172/jci.insight.153471 (PMC9714792; doi:10.1172/jci.insight.153471)
Supplement: Supplemental data [file jciinsight-7-153471-s107.pdf]

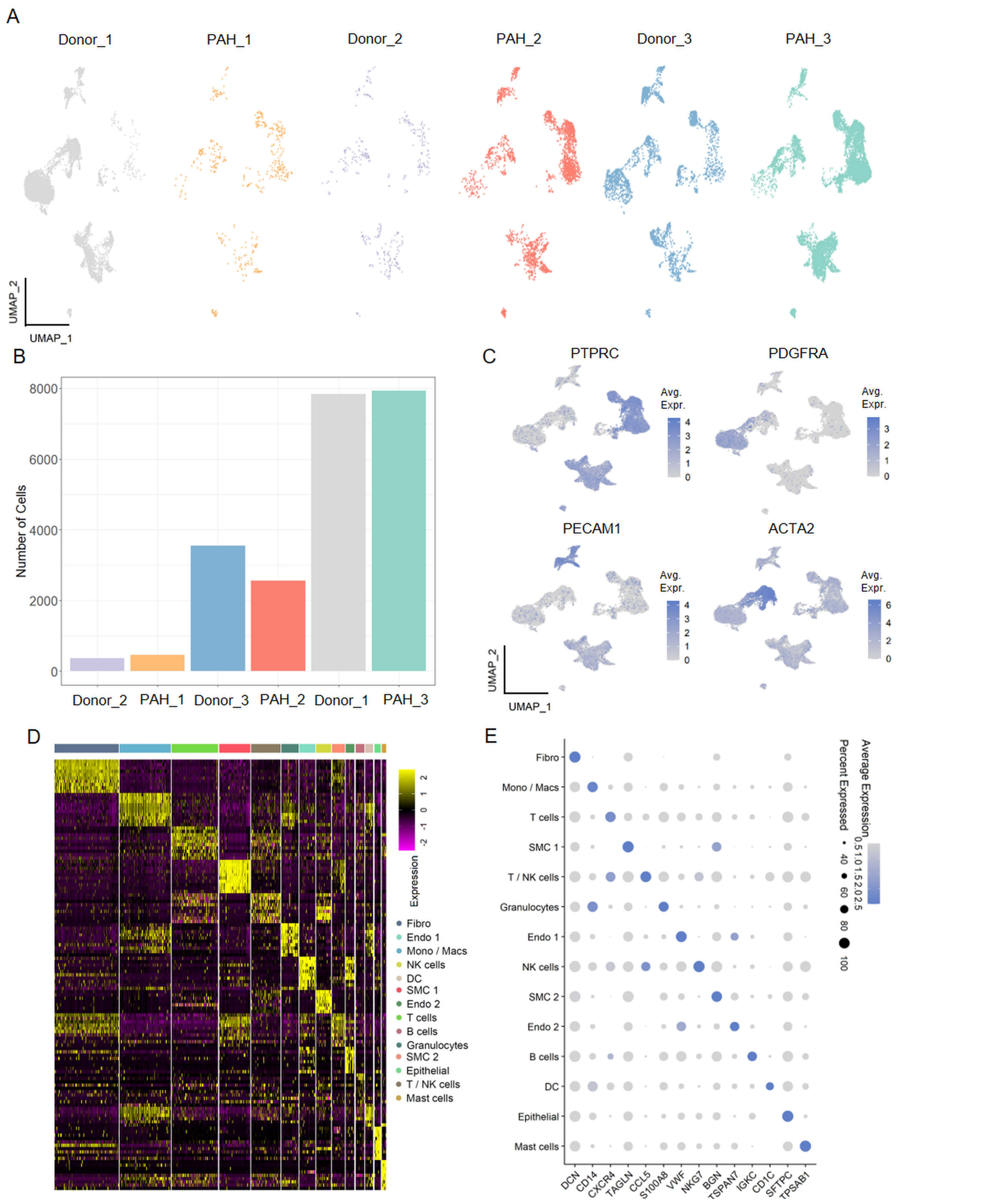

Supplemental Figure 1. Integration of human pulmonary artery single-cell RNA-sequencing data

(**A**) Uniform Manifold Approximation and Projection (UMAP) overlay of each single donor and pulmonary arterial hypertension (PAH) single-cell RNA-sequencing (scRNAseq) sample. (**B**) Histogram showing number of captured and analysed cells for each donor and PAH sample. (**C**) UMAP gene expression plots of major cell type markers: Protein Tyrosine Phosphatase Receptor Type C (*PTPRC*) for immune cells (CD45), Platelet Derived Growth Factor Receptor Alpha (*PDGFRA*) for fibroblasts, Platelet and Endothelial Cells Adhesion Molecule 1 (*PECAM1*) for endothelial cells (CD31), and Alpha Smooth Muscle Actin 2 (*ACTA2*) for smooth muscle cells. The color gradient represents the average expression across the human pulmonary artery scRNAseq dataset. (**E**) Heatmap of top 10 enriched genes in each cluster. Wilcoxon rank sum test with Bonferroni adjustment, $p<0.05$ and $|\log_2(\text{fold change})|>0.25$. (**F**) Dot plot of cluster-identifying genes. Dot size represents percentage of cells expressing the gene, color gradient represents the average expression.

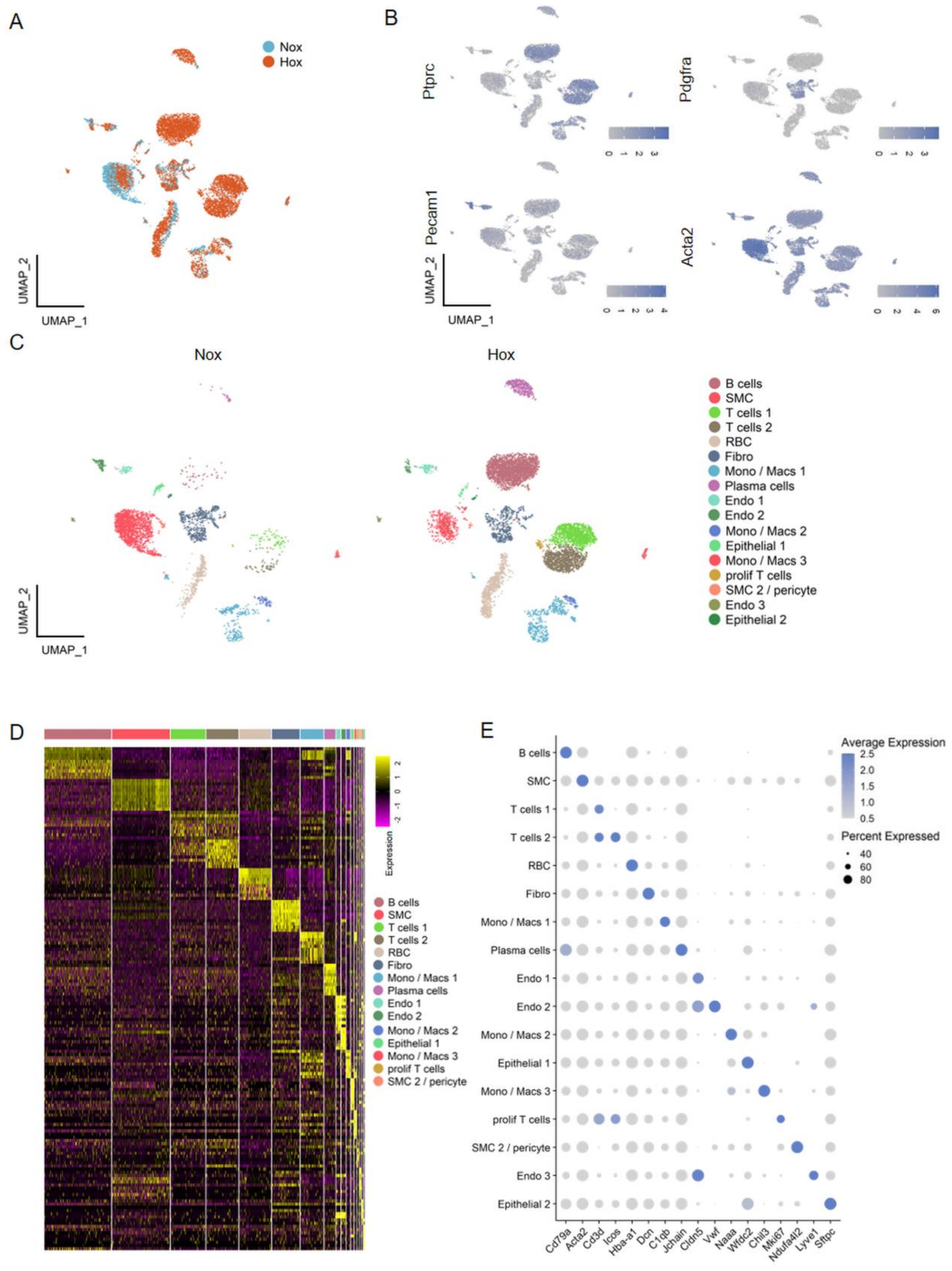

Supplemental Figure 2. Integration of murine pulmonary artery single-cell RNA-sequencing data

(**A**) Uniform Manifold Approximation and Projection (UMAP) of the single-cell RNA-sequencing (scRNAseq) data from murine pulmonary artery (PA) and labelled by experimental condition. Single cell capture was done on a pooled sample from n=3 mice for each condition (normoxia, 3 weeks hypoxia). (**B**) UMAP gene expression plots of major cell type markers: Protein Tyrosine Phosphatase Receptor Type C (*Ptprc*) for immune cells (CD45), Platelet Derived Growth Factor Receptor Alpha (*Pdgfra*) for fibroblasts, Platelet and Endothelial Cells Adhesion Molecule 1 (*Pecam1*) for endothelial cells (CD31), and Alpha Smooth Muscle Actin 2 (*Acta2*) for smooth muscle cells. The color gradient represents the average expression across the human pulmonary artery scRNAseq dataset. (**C**) UMAP of murine PA scRNAseq data with cluster annotation (B cells = B cells, SMC = Smooth Muscle Cells, RBC = Red Blood Cells, Fibro = Fibroblasts, Mono / Macs 1,2,3 = Monocytes and Macrophages 1,2,3, Endo 1,2,3 = Endothelial cells 1,2,3 prolif T cells = Proliferating T cells, SMC 2 / Pericytes = Smooth Muscle Cells 2 and Pericytes). (**D**) Heatmap of top10 enriched genes in each cell cluster. Wilcoxon rank sum test with Bonferroni adjustment, $p<0.05$ and $|\log_2(\text{fold change})|>0.25$. (**E**) Dot plots showing expression profiles of highest cluster-enriched genes. Dot size represents percentage of cells expressing the gene, color gradient represents the average expression across the whole dataset.

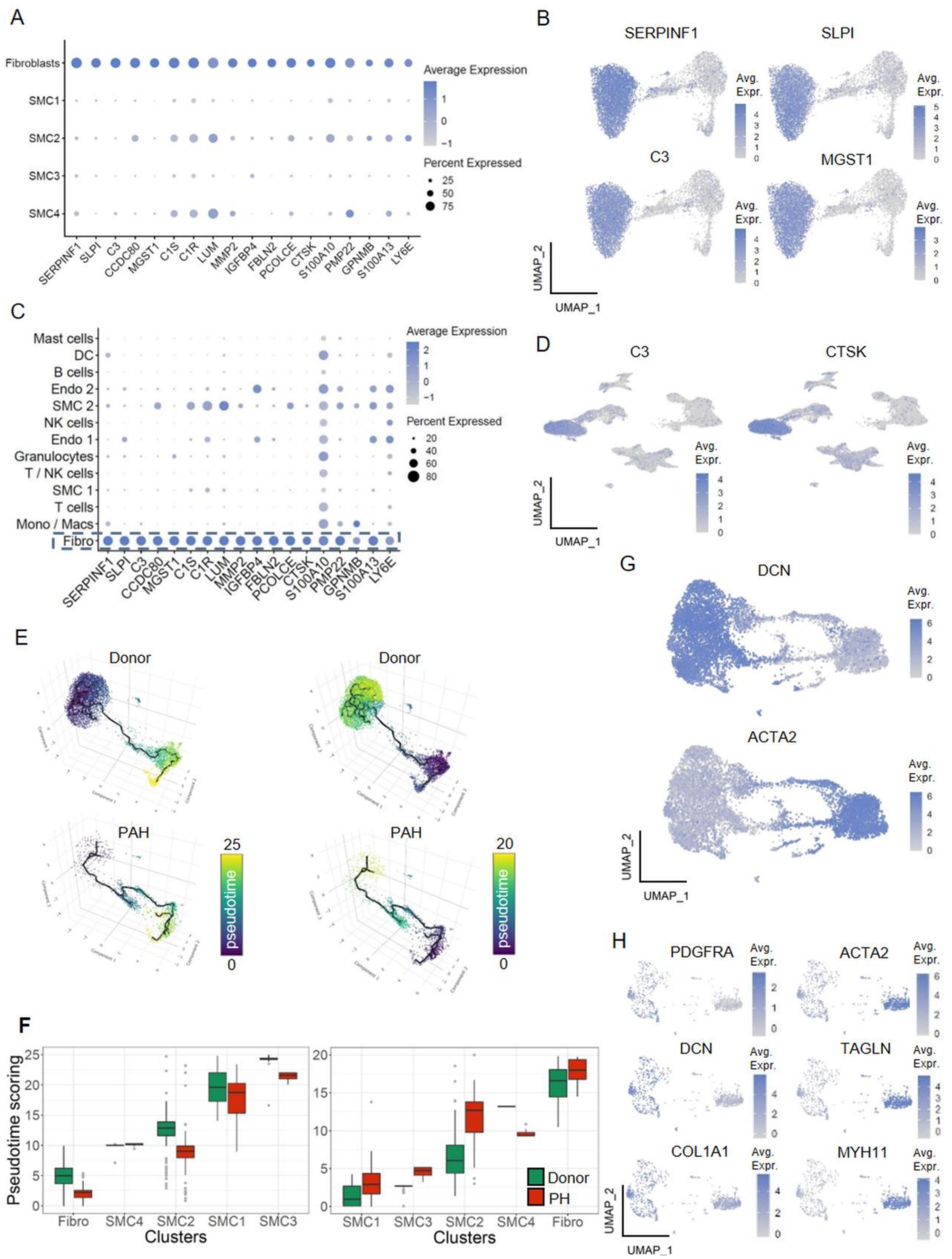

Supplemental Figure 3. Distinguishing features of fibroblast, intermediary and smooth muscle cell clusters

(**A**) Dot plots showing expression profile of top 18 genes enriched in fibroblast cluster compared to all smooth muscle cell (SMC) clusters. Dot size represents percentage of cells expressing the gene, the color gradient represents average expression across the whole dataset. Wilcoxon rank sum test with Bonferroni adjustment, $p<0.05$ and $|\log_2(\text{fold change})|>0.25$. (**B**) Uniform Manifold Approximation and Projection (UMAP) expression plots of four markers (*SERPINF1*, *SLPI*, *C3,* and *MGST1*) with high discriminatory power between fibroblasts and smooth muscle cell clusters. The color gradient represents the average expression across the human fibroblasts and SMC data subset. (**C**) Dot plots showing expression profile of top 18 genes enriched in fibroblast cluster compared to the rest of PA clusters. Dot size represents percentage of cells expressing the gene, color gradient represents the average expression across the human pulmonary artery (PA) dataset. (**D**) UMAP expression plots of two markers (*CTSK* and *C3*) with high discriminatory power between fibroblasts and the rest of PA clusters. The color gradient represents the average expression across the human PA dataset. (**E**) Color-coded pseudotime calculation overlayed on 3D UMAP of the SMC and fibroblast subset using fibroblasts (on the left) and SMC (on the right) as root nodes. (**F**) Quantification of the pseudotime scoring represented as a boxplot (Donor in green and PAH in red) with fibroblast as root node (left panel) and SMC1 as root node (right panel). (**G**) UMAP expression plot of *DCN/Dcn* and *ACTA2/Acta2* on integrated human and murine SMC-fibroblast PA subset. The color gradient represents the average gene expression across the data subset. (**H**) UMAP expression plots of classical SMC markers (*ACTA2/Acta1*, *TAGLN/Tagln, MYH11/Myh11*) and fibroblast markers (*PDGFRA/Pdgfra*, *DCN/Dcn*, *COL1A1/Col1a1*). The color gradient represents the average expression across the murine SMC-fibroblast subset from integrated human-murine PA scRNAseq dataset.

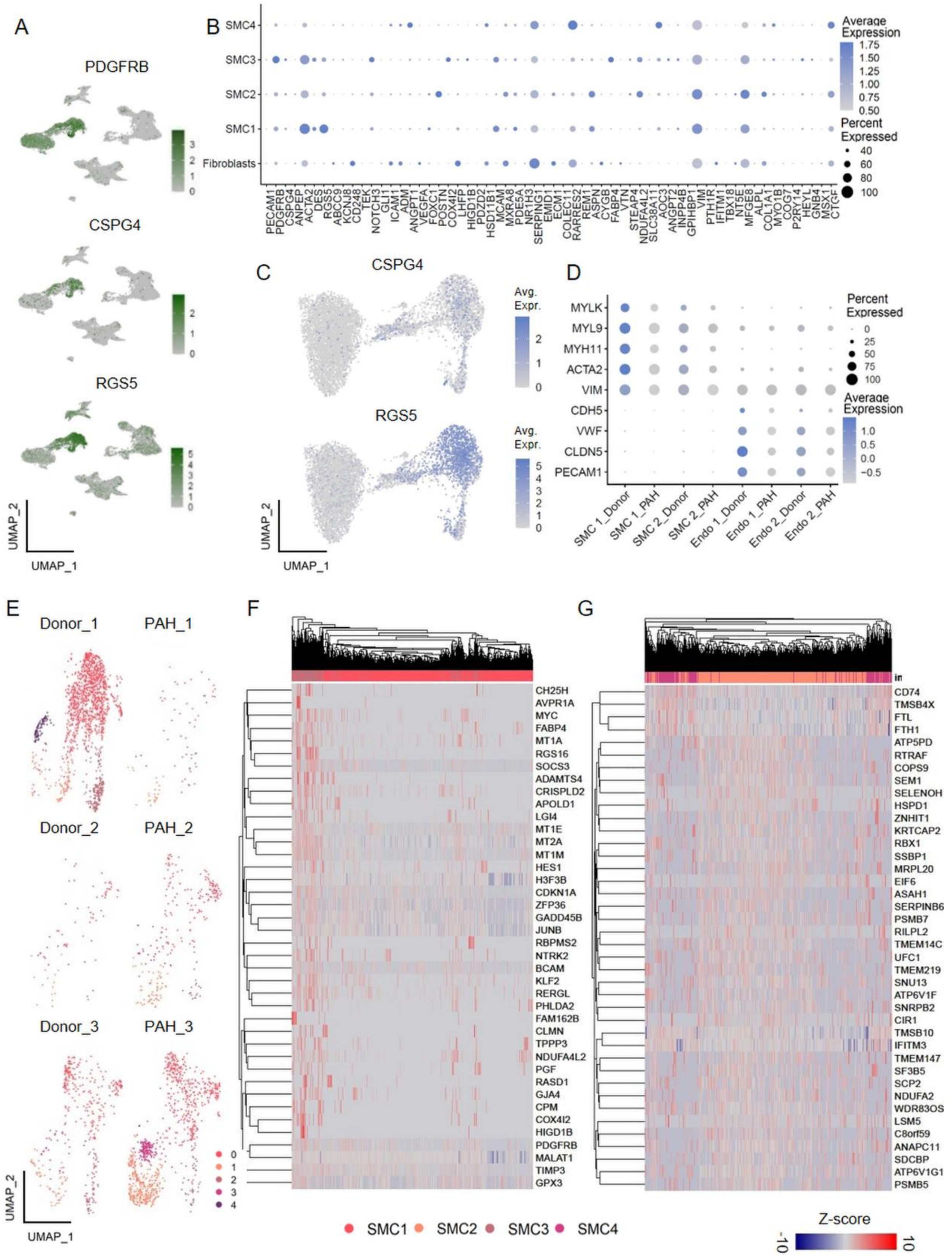

Supplemental Figure 4. Distinguishing features of smooth muscle cell clusters

(**A**) Uniform Manifold Approximation and Projection (UMAP) expression plots of three classical pericyte markers: *PDGFRB*, *CSPG4* and *RGS5*. The color gradient represents average

expression across the entire human pulmonary artery (PA) dataset. (**B**) Dot plots showing expression profile of genes enriched in pericytes according to the PangladoDB database. Dot size represents percentage of cells expressing the gene, color gradient represents average expression across the SMC-fibroblast data subset. (**C**) UMAP expression plots of *CSPG4* and *RGS5*. The color gradient represents the average expression across the SMC-fibroblast data subset. (**D**) Dot plots showing expression profile of SMC and endothelial marker genes. Dot size represents percentage of cells expressing the gene, color gradient represents average expression across the SMC and endothelial clusters in human PA dataset and separated according to condition. (**E**) UMAP of SMC clusters for each single donor and pulmonary arterial hypertension (PAH) single-cell RNA-sequencing (scRNAseq) sample. Hierarchical clustering heatmap showing top 40 differnetially expressed genes between SMC1 compared to SMC3 (**F**), and SMC2 compared to SMC4 (**G**). The color gradient was inferred according to z-score. Wilcoxon rank sum test with Bonferroni adjustment, $p<0.05$ and $|\log_2(\text{fold change})|>0.1$.

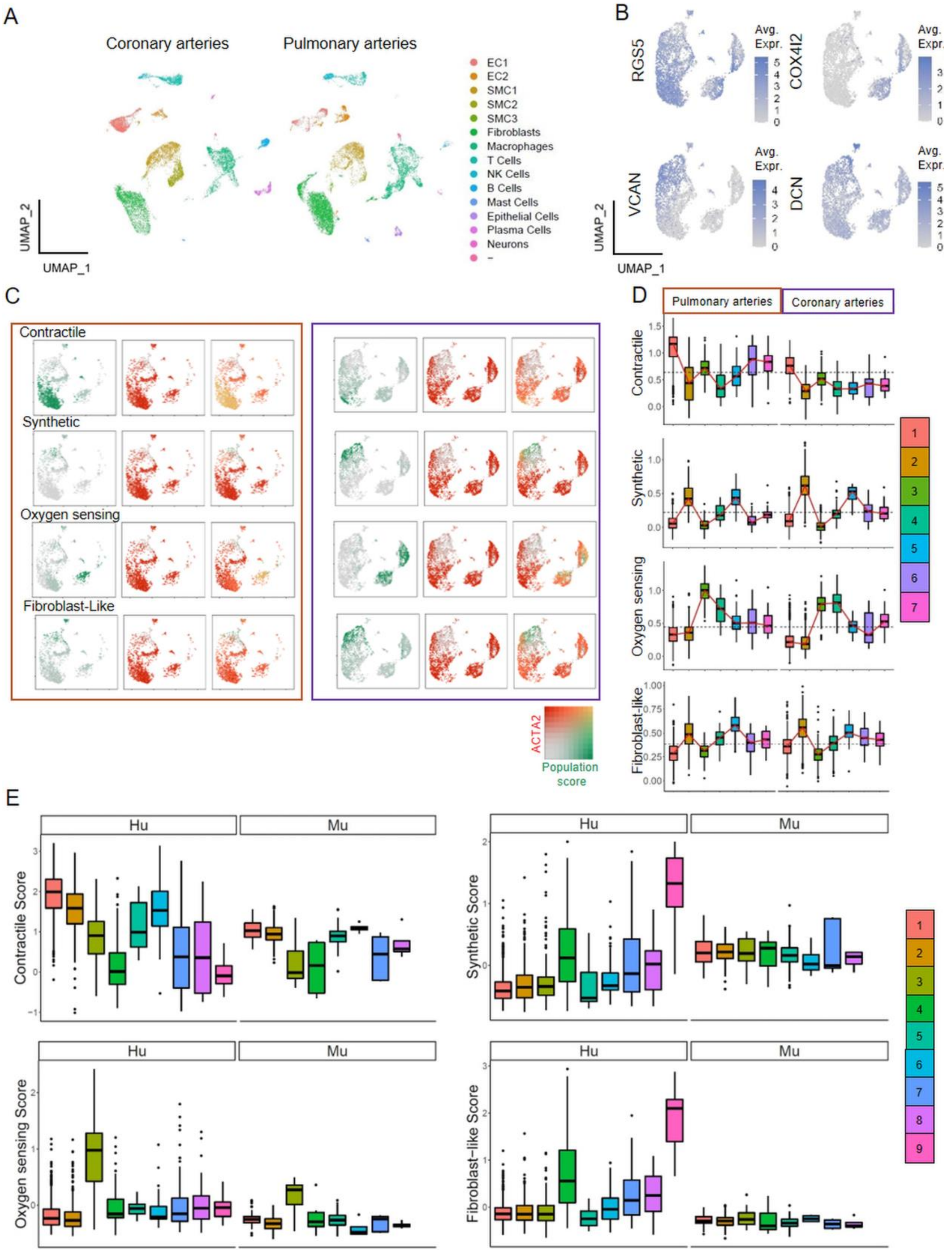

Supplemental Figure 5. Cross-compartment and species comparison of arterial smooth muscle cell heterogeneity

(**A**) Uniform Manifold Approximation and Projection (UMAP) of the concatenated human pulmonary artery (PA) and coronary artery (CA) single-cell RNA-sequencing dataset (EC1,2 = Endothelial cells 1,2, SMC1,2,3 = Smooth Muscle Cells 1,2,3. (**B**) UMAP expression plots of the PA SMC cluster-enriched markers. *RGS5*, for contractile SMC, *VCAN*, for synthetic SMC, *COX4I2*, for oxygen sensing SMC, and *DCN* for fibroblast-like SMC. The color gradient represents average expression across the integrated SMC data subset from the concatenated PA-CA human dataset. (**C**) UMAP expression plots of the top 100 PA-SMC cluster-enriched genes, inferred as subpopulations specific score (green), overlayed with the SMC marker *ACTA2* (red) in the integrated SMC data subset from the concatenated PA-CA human dataset and separated according to original dataset. Wilcoxon rank sum test with Bonferroni adjustment, $p<0.05$ and $|\log_2(\text{fold change})|>0.25$. The color gradient was inferred by calculating the average expression of the subpopulation specific genes query (top 100 PA-SMC cluster-enriched genes) and then subtract the average expression of an equivalent set of randomly selected control genes across the dataset. (**D**) Boxplot visualizing SMC cluster-specific score in each cluster of the integrated SMC data subset from the concatenated PA-CA human dataset and separated according to original dataset. Dashed line represents the mean of the subpopulation score across the integrated dataset. (**E**) Boxplot visualizing SMC cluster-specific score in each cluster of the integrated SMC data subset from the concatenated human-murine PA dataset separated according to species.

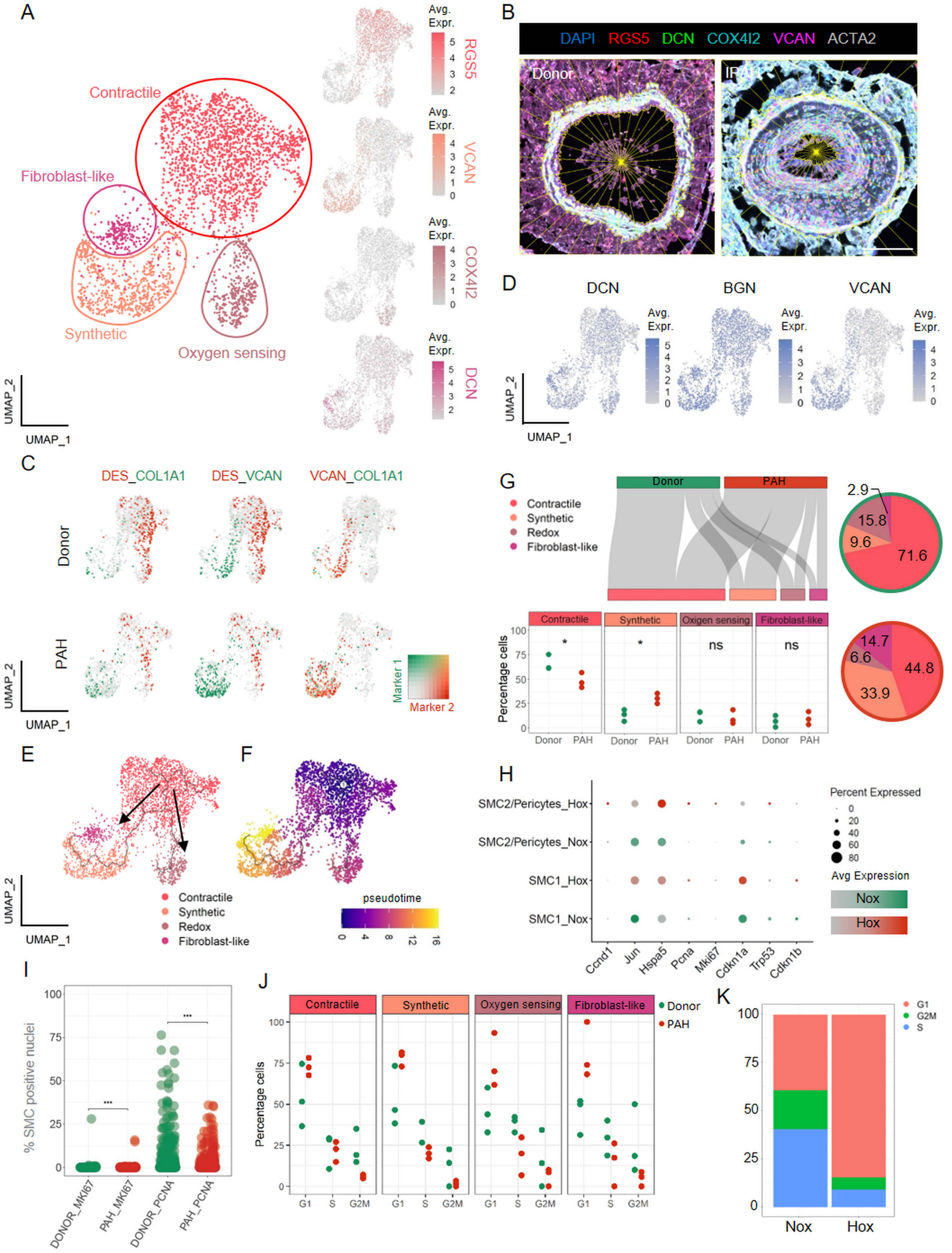

Supplemental Figure 6. Dynamic changes between smooth muscle cell clusters upon pulmonary vascular remodeling

(**A**) Annotated Uniform Manifold Approximation and Projection (UMAP) (on the left) and UMAP expression plot of smooth muscle cell (SMC) cluster markers: *RGS5* (contractile SMC), *VCAN* (synthetic SMC), *COX4I2* (oxygen sensing SMC) and *DCN* (fibroblast-like SMC). The color gradient represents the average expression across the SMC subset. (**B**) Representative images depicting the automated, unbiased analysis of SMC cluster marker localization from donor pulmonary arteries (n=4/ PA=7) and idiopathic pulmonary arterial hypertension (IPAH, n=5, PA=7), diameter range 50-200μm. Each image was overlaid with 32 equally spaced probes automatically set and measured, following an algorithm-based detection of the vessel. The luminal center of gravity is used as the origin for the probes. (**C**) UMAP expression plots of contractile and synthetic markers in the SMC data subset: *DES* (red) and *COL1A1* (green, left panel). *DES* (red) and *VCAN* (green, middle panel). *VCAN* (red) and *COL1A1* (green, right panel). The color gradient represents the average expression across the SMC data subset. (**D**) UMAP expression plots of *DCN*, *BGN* and *VCAN*. The color gradient represents the average expression across the SMC subset. (**E**) Trajectory inference overlayed on UMAP of the SMC subset. Arrows describe the two hypothesized routes. (**F**) Color coded pseudotime calculation overlayed on UMAP of the SMC subset using contractile SMC as root node. (**G**) Sankey diagram (top panel), pie charts (right panel) and percentage (lower panel) showing distribution of SMC among clusters and condition (donor, PAH). T-test, $p<0.05$. (**H**) Dot plot expression profile of eight proliferation-related genes. Dot size represents percentage of cells expressing the gene, color gradient represents the average expression across the SMC subset. (**I**) Percentage of *ACTA2*-positive nuclei for *PCNA* and *MKI67*. Wilcoxon rank sum test, $p<0.05$. (**J**) Percentage of cells belonging to G1, G2M and S phase in the SMC dataset per sample. (**K**) Proportions of cells in G1, G2M or S phase in the SMC murine PA. The cell phase score was inferred by subtracting the average expression of cell cycle specific genes and the average expression of an equivalent set of randomly selected control genes.
