# Supplementary material for: Single-cell transcriptomics reveals skewed cellular communication and phenotypic shift in pulmonary artery remodeling
Source: JCI Insight. 2022 Oct 24;7(20):e153471. doi: 10.1172/jci.insight.153471 (PMC9714792; doi:10.1172/jci.insight.153471)
Supplement: Supplemental table 8 [file jciinsight-7-153471-s115.pdf]

Supplemental Table 8: Patient characteristics.

Diagnosis	Donor_1	PAH_1	Donor_2	PAH_2	Donor_3	PAH_3
**Age**	54	37	40	52	56	63
**Sex**	M	M	M	F	M	M
**Height (cm)**		180	170	169	180	174
**Weight (kg)**		88	65	76	85	71
**BMI**		27.2	22.5	26.6	26.2	23.5
**6MWD**		270		275		
**FEV1/FVC%**		75.8				45.1
**FEV1%**		45.0		79.8		47.0
**DLCO cSB%**		47.9		51.0		11.2
**pO2 (mmHg)**		60.5	105.0	60.0	94.0	37.1
**pCO (mmHg)**		32.5	42.3	30.0	33.0	32.8
**NTproBNP (pg/ml)**		2886		458.1		177.8
**mPAP (mmHg)**		71.0		77.0		68.0

BMI = body mass index, 6MWD = 6 minute walk distance, FEV1 = forced expiratory volume in 1 second, FVC = forced vital capacity, DLCO cSB = single breath diffusing capacity of lung for carbon monoxide corrected for hemoglobin, pO2 = partial pressure of O2, pCO = partial pressure of CO, NTproBNP = N-terminal pro-brain natriuretic peptide, mPAP = mean pulmonary arterial pressure.
